# Antenatal blood pressure for prediction of pre-eclampsia, preterm birth, and small for gestational age babies: development and validation in two general population cohorts

**DOI:** 10.1136/bmj.h5948

**Published:** 2015-11-17

**Authors:** Corrie Macdonald-Wallis, Richard J Silverwood, Bianca L de Stavola, Hazel Inskip, Cyrus Cooper, Keith M Godfrey, Sarah Crozier, Abigail Fraser, Scott M Nelson, Debbie A Lawlor, Kate Tilling

**Affiliations:** 1 MRC Integrative Epidemiology Unit at the University of Bristol, Bristol BS8 2BN, UK; 2 School of Social and Community Medicine, University of Bristol, Bristol BS8 2BN, UK; 3 Department of Medical Statistics, London School of Hygiene and Tropical Medicine, London WC1E 7HT, UK; 4 Centre for Statistical Methodology, London School of Hygiene and Tropical Medicine, London WC1E 7HT, UK; 5 MRC Lifecourse Epidemiology Unit, University of Southampton, Southampton General Hospital, Southampton SO16 6YD, UK; 6 NIHR Southampton Biomedical Research Centre, University of Southampton and University Hospital Southampton NHS Foundation Trust, Southampton General Hospital, Southampton SO16 6YD, UK; 7 National Institute for Health Research Musculoskeletal Biomedical Research Unit, University of Oxford, Oxford OX3 7LE, UK; 8 School of Medicine, University of Glasgow, Glasgow G12 8QQ, UK

## Abstract

**Study question** Can routine antenatal blood pressure measurements between 20 and 36 weeks’ gestation contribute to the prediction of pre-eclampsia and its associated adverse outcomes?

**Methods** This study used repeated antenatal measurements of blood pressure from 12 996 women in the Avon Longitudinal Study of Parents and Children (ALSPAC) to develop prediction models and validated these in 3005 women from the Southampton Women’s Survey (SWS). A model based on maternal early pregnancy characteristics only (BMI, height, age, parity, smoking, existing and previous gestational hypertension and diabetes, and ethnicity) plus initial mean arterial pressure was compared with a model additionally including current mean arterial pressure, a model including the deviation of current mean arterial pressure from a stratified normogram, and a model including both at different gestational ages from 20-36 weeks.

**Study answer and limitations** The addition of blood pressure measurements from 28 weeks onwards improved prediction models compared with use of early pregnancy risk factors alone, but they contributed little to the prediction of preterm birth or small for gestational age. Though multiple imputation of missing data was used to increase the sample size and minimise selection bias, the validation sample might have been slightly underpowered as the number of cases of pre-eclampsia was just below the recommended 100. Several risk factors were self reported, potentially introducing measurement error, but this reflects how information would be obtained in clinical practice.

**What this study adds** The addition of routinely collected blood pressure measurements from 28 weeks onwards improves predictive models for pre-eclampsia based on blood pressure in early pregnancy and other characteristics, facilitating a reduction in scheduled antenatal care.

**Funding, competing interests, data sharing** UK Wellcome Trust, US National Institutes of Health, and UK Medical Research Council. Other funding sources for authors are detailed in the full online paper. With the exceptions of CM-W, HMI, and KMG there were no competing interests.

## Introduction

Pre-eclampsia is the most severe form of hypertensive disorder of pregnancy and is associated with maternal and fetal mortality,^1^ ^2^ intrauterine growth restriction, and preterm birth.^3^ ^4^ ^5^ It is defined as de novo high blood pressure occurring after 20 weeks’ gestation in conjunction with proteinuria,^6^ but these symptoms can develop swiftly, within days of a normal antenatal assessment, and deficiencies in care have previously been reported in relation to maternal and fetal deaths attributable to pre-eclampsia in the United Kingdom.^7^ ^8^ Although there is no known cure for pre-eclampsia other than delivery of the baby,^9^ decisions such as whether to expectantly monitor or to deliver are important for both maternal and fetal outcome.^10^ Therefore it is important to be able to identify women at risk as early in pregnancy as possible to allow for adequate surveillance and early detection of disease.^8^ We,^11^ and others,^12^ have shown that before the diagnostic threshold for pre-eclampsia is met, the pattern of change in blood pressure during pregnancy differs between women who remain normotensive and those who subsequently develop pre-eclampsia. Specifically women who go on to experience pre-eclampsia have a higher initial blood pressure and a steeper increase after the mid-pregnancy nadir. A greater increase in blood pressure across pregnancy is also associated with a shorter gestation and a lower birthweight baby.^4^ ^13^ ^14^

Several prediction models have shown the value of using blood pressure recorded at the first visit to the antenatal clinic alongside other maternal characteristics to predict the later development of pre-eclampsia.^15^ ^16^ ^17^ ^18^ ^19^ ^20^ ^21^ ^22^ As blood pressure is routinely measured on repeated occasions during pregnancy in most high income countries, and could also be feasibly measured in low and middle income countries, the incorporation of subsequent measurements and the trajectory of change in blood pressure in clinical prediction models could add additional value and improve the information available to make decisions about care. As well as identification of women at high risk who would benefit from intensive monitoring to prevent the adverse outcomes associated with pre-eclampsia, it might also be beneficial to be able to identify a group of women at low risk and transfer them to a reduced schedule of antenatal appointments.^23^

We have previously developed stratified normal reference ranges for blood pressure, which allow for a different expected trajectory of blood pressure for different subgroups of women.^24^ In this study we assessed and externally validated the predictive ability of blood pressure measurements at different gestational ages, and their comparison with our reference ranges, to identify women at higher and low risk of pre-eclampsia using two prospective cohort studies from the UK. We additionally investigated whether such measurements were able to identify pregnancies at risk of preterm birth and small for gestational age babies, as these are two of the perinatal outcomes that are most commonly associated with pre-eclampsia.

This paper conforms to TRIPOD guidelines.^25^

## Methods

### Avon Longitudinal Study of Parents and Children (ALSPAC)

For the development of the prediction models we used the Avon Longitudinal Study of Parents and Children (ALSPAC), a prospective birth cohort study that recruited women with expected delivery dates between 1 April 1991 and 31 December 1992 living in a defined area of Avon during pregnancy. The study has been described in full elsewhere,^26^ and the study website contains details of available data (www.bris.ac.uk/alspac/researchers/data-access/data-dictionary). A total of 14 541 women were enrolled, 13 678 had a singleton pregnancy resulting in a live birth, and data on measurements of blood pressure were available for 12 996 of these women.

#### Obstetric measurements in ALSPAC

Six trained research midwives abstracted all measurements of blood pressure and proteinuria that were taken as part of routine antenatal care by midwives or obstetricians from the women’s obstetric records. There was no variation between midwives in mean values of the data abstracted, and error rates were consistently <1% in repeated data entry checks. Measurements were taken once in women in the seated position with the appropriate cuff size, with Korotkoff phase V used for diastolic blood pressure.

#### Maternal and offspring characteristics in ALSPAC

Maternal age at delivery and sex of the baby were obtained from obstetric records. Maternal weight and height before pregnancy, together with parity, smoking status, education, hypertension, and diabetes before the index pregnancy and ethnicity and household social class were obtained from questionnaires administered during pregnancy. Smoking during pregnancy was classed as “never” or “any smoking” for women who reported smoking either in the first three months of pregnancy or within two weeks of the 18 week questionnaire. Women who reported ever having hypertension outside of pregnancy were classed as having essential hypertension, while women who reported having hypertension only in pregnancy were classed as having previous gestational hypertension. Similarly, women who reported having ever had diabetes outside of pregnancy were defined as having diabetes while women who reported having diabetes only in a previous pregnancy were defined as having previous gestational diabetes.

### Southampton Women’s Survey (SWS)

In accordance with recent guidelines on the development and assessment of prognostic models,^27^ we externally validated our prediction models in a further dataset. The Southampton Women’s Survey is a prospective cohort study that recruited non-pregnant women through general practices in Southampton between April 1998 and September 2002. Full details of the study have been published previously.^28^ The study enrolled 12 583 women, of whom 3158 became pregnant and had delivered a singleton liveborn infant by the end of 2007. Obstetric blood pressure measurements were available for 3005 of these.

#### Obstetric measurements in SWS

A team of trained research midwives abstracted all measurements of blood pressure that were routinely collected during pregnancy from the women’s obstetric records after delivery following a strict protocol. The measurements were ordered by time, and the data checked for consistency.

#### Maternal and offspring characteristics in SWS

Maternal age at delivery and sex of offspring were obtained from obstetric records. Information on maternal parity, education, social class, and ethnicity was obtained at the initial interview before pregnancy, and height was measured. At the 11 week interview the mothers reported pre-pregnancy weight, any diabetes in a previous pregnancy or outside of pregnancy, and high blood pressure in a previous pregnancy or outside of pregnancy. Smoking status was obtained during interviews at 11 and 34 weeks’ gestation and classified as “never smoked in pregnancy” or “any smoking in pregnancy.” An algorithm that incorporated information recorded before pregnancy and at six months postpartum was used to estimate smoking in pregnancy when data at one or both time points were missing.

#### Initial blood pressure and gestational age specific blood pressure in ALSPAC and SWS

As preliminary analysis suggested that mean arterial pressure was a stronger predictor of each outcome than systolic blood pressure (SBP) or diastolic blood pressure (DSP) separately, we used this as our measure of blood pressure in prediction models. This was defined as (SBP+2×DBP)/3. In both cohorts, the initial measurement of mean arterial pressure was defined as the first antenatal measurement, provided that this occurred before 18 weeks’ gestation. We also derived mean arterial pressure at 20, 25, 28, 31, 34, and 36 weeks’ gestation for each woman (following the standard schedule of antenatal appointments for nulliparous women used in the UK^29^), using the measurement that was closest to this gestational age and within two weeks of it, or classifying the variable as missing if there was no measure within this gestational age range.

#### Pregnancy outcomes in ALSPAC and SWS

In ALSPAC pre-eclampsia was defined by the International Society for the Study of Hypertension in Pregnancy (ISSHP) criteria^6^ as systolic blood pressure ≥140 mm Hg and/or diastolic blood pressure ≥90 mm Hg along with proteinuria of ≥1+ on urine dipstick testing occurring on two occasions after 20 weeks’ gestation. In SWS we used information on any diagnosis of pre-eclampsia that was recorded in obstetric records to define preeclampsia. In both cohorts, we defined small for gestational age as below the 10th centile of birth weight adjusted for gestational age at birth using internal standardisation by cohort. In ALSPAC the final clinical estimate of the expected date of delivery was abstracted from the obstetric records and used to calculate gestational age at delivery. In SWS we used a computerised algorithm to derive gestational age using menstrual data (66%) or, when these were uncertain or discrepant with ultrasound assessments, fetal anthropometry in early pregnancy. Preterm birth was defined as birth before 37 weeks’ gestation.

Table A in appendix 1 gives a summary of definitions of all outcomes and predictor variables in ALSPAC and SWS for comparison.

### Statistical analysis

#### Deriving expected trajectories of mean arterial pressure for each woman

In the first stage of model development we developed expected trajectories of mean arterial pressure across pregnancy, or normograms, in ALSPAC, similarly to those described previously.^24^ For the 9402 women in ALSPAC who had a normal pregnancy without pre-eclampsia, essential hypertension, or diabetes, and delivered a term infant appropriate size for gestational age we used multilevel models to describe the trajectory of change in mean arterial pressure using the observed repeated measurements across the whole of pregnancy and the exact gestational ages at measurement. The multilevel models had two levels: measurement occasion (level 1) within individual (level 2). We used restricted cubic splines with knots at 11, 18, 30, 36, and 40 weeks’ gestation to describe the shape of the trajectory as this was found to have the best fit to these data in previous analysis.^24^ We allowed for different average trajectories according to BMI category and smoking status by including appropriate interaction terms and fitted separate models for nulliparous and multiparous women.

For all women in the ALSPAC cohort (including non-normal pregnancies) we then obtained a normative value of mean arterial pressure at 20, 25, 28, 31, 34, and 36 weeks’ gestation, which accounted for their parity and BMI before pregnancy and whether they smoked during pregnancy, and was conditional on their mean arterial pressure at their initial visit (before 18 weeks’ gestation). Appendix 2 gives further information on the derivation of this normative value.

#### Missing data

For both the ALSPAC and SWS datasets there were missing data on maternal characteristics and also for the derived values of initial mean arterial pressure and mean arterial pressure at 20, 25, 28, 31, 34, and 36 weeks for some women. To increase power and minimise selection bias in the second stage prediction models, we next used multivariable multiple imputation of missing data. Separate imputation models were used for the two cohorts, imputing to the full 12 996 women in ALSPAC and 3005 women in SWS who had any measurements of mean arterial pressure in pregnancy. For each imputation model, we included all early pregnancy characteristics and derived mean arterial pressure values as well as the outcomes. We imputed any missing data for the normative values of mean arterial pressure passively from imputed early pregnancy characteristics and initial mean arterial pressure. For each imputation we generated 20 imputed datasets and combined coefficient estimates across these using Rubin’s rules.^30^ ^31^ Imputation of missing data in this way assumes that data are missing at random—that is, that any reasons for missingness are explained by the observed variables included in the imputation model.^32^ The assumption seems reasonable here as most women will not be aware of their current blood pressure (only their previous values) so this is unlikely to influence their decision to have their blood pressure measured at any given gestational age. Full details of the imputation models are included in appendix 2.

#### Prediction of pre-eclampsia, preterm birth, and small for gestational age

In the second stage we used logistic regression to obtain separate prediction models for pre-eclampsia, preterm birth, and small for gestational age in ALSPAC based on the imputed datasets. Maternal predictors were selected from BMI before pregnancy, height, age ≥35, parity, smoking, essential hypertension, previous gestational hypertension, diabetes, previous gestational diabetes, and non-white ethnicity. This set of predictors was chosen because of strong prior evidence of their association with pre-eclampsia, ease of measuring in clinical practice, and generalisability to different settings. We used backward selection, with a P value threshold of 0.2, to select variables for inclusion in these early pregnancy characteristic models. Mean arterial pressure at the initial visit was forced to be included in all of these prediction models. The early pregnancy characteristic model was called model 1. We then added mean arterial pressure at gestational ages 20, 25, 28, 31, 34, or 36 weeks (each tested separately) to determine the added value of such measurements from 20 weeks in predicting outcomes (model 2). In model 3 we added the difference between the woman’s measurement of mean arterial pressure at each gestational age and their normative value (that is, their deviation from their expected trajectory for their BMI, parity, smoking status, and initial mean arterial pressure) to model 1. Finally, model 4 combined models 2 and 3 by adding both the value of mean arterial pressure and the deviation from the normative value at each gestational age to model 1. Table B in appendix 1 gives a summary of variables included in each model. The areas under the receiver operating characteristics curves (ROCs) of the models were compared with a Wald test based on a bootstrapped standard error for the difference between areas. For predictions at each gestational age, we included in prediction models only women who had not yet delivered and aimed to predict the development of pre-eclampsia at any stage of pregnancy, any subsequent preterm births, and small for gestational age offspring measured at birth. Table C in appendix 1 shows the cumulative number of women who had delivered at each gestational age. We also considered including all measurements of mean arterial pressure up to a given gestational age in prediction models rather than just the initial and the current measure but found no meaningful improvement in the area under the curve.

The parameters from the first stage multilevel model were then used to derive normative values of mean arterial pressure at each gestational age in SWS and these were combined with parameters from the second stage logistic regression model developed in ALSPAC and applied to women in the SWS cohort to externally validate our prediction model. The model parameters for all outcomes are given in appendix 2.

We assessed the predictive ability of these models using the area under the receiver operating characteristic curve and the sensitivity, specificity, and positive and negative predictive values, together with positive and negative likelihood ratios. We assessed calibration by comparing the actual versus the predicted risk across 10ths of the risk score for each outcome. The prediction models were recalibrated for the SWS when necessary by adjusting the intercept and slope of the logistic regression model for this cohort (see appendix 2).^33^

We assessed the expected numbers of women out of an initial cohort of 1000 who would be classified as low risk and high risk if we were to use a sequential screening strategy to rule out pre-eclampsia at each gestation considered up to 31 weeks. Model 1 was used at booking, followed by model 2 at 20, 25, 28, and 31 weeks. We tested two thresholds for the screening strategy (applying the same threshold of the risk score at each gestational age of testing), which were derived from the average threshold (across all gestational ages from 20 to 36 weeks) required to give sensitivities of 95% and 99%, respectively, at each gestational age in ALSPAC. These thresholds were then applied to the SWS to produce a flowchart of the expected numbers of women in each group at each stage of testing.

All analyses were completed in Stata version 13.1 and multilevel models were fitted using MLwiN version 2.30 through Stata using the runmlwin command.^34^

### Patient involvement

This paper is based on two prospective cohort studies that collect data from members of the general population (not patients) on a large number of potential risk factors and health outcomes and that are used widely by the research community to examine a wide range of health related questions. Consequently no patients were involved in setting the research question or the outcome measures, nor were they involved in recruitment or the design and implementation of the study. There are no plans to involve patients in dissemination.

## Results

Table 1 shows maternal and pregnancy related characteristics in the imputed data pre-eclampsia status.[Table tbl1] Tables D and E in appendix 1 compare their distributions with observed data. Characteristics were generally similar in the imputed data compared with the observed data. Women in the SWS were more likely to be overweight or obese, to be nulliparous, to have had previous gestational hypertension or gestational diabetes, and to be of non-white ethnicity and were less likely to smoke or to have pre-existing hypertension, were shorter and older on average, and had higher initial mean arterial pressure than women in the ALSPAC cohort.

**Table 1 tbl1:** Characteristics of pregnancies in Avon Longitudinal Study of Parents and Children (ALSPAC) and Southampton Women’s Survey (SWS) by pre-eclampsia status using imputed datasets

	ALSPAC				SWS		
	Without pre-eclampsia (n=12 679)	With pre-eclampsia (n=317)	Overall (n=12 996)		Without pre-eclampsia (n=2917)	With pre-eclampsia (n=88)	Overall (n=3005)
Mean (SD; range for overall mean) mean arterial pressure:							
Initial	81.2 (8.7)	87.0 (10.2)	81.3 (8.8; 47-130)		81.8 (8.6)	85.5 (11.2)	81.9 (8.7; 55-123)
At 20 weeks	80.1 (8.4)	87.2 (10.3)	80.3 (8.5; 45-130)		80.1 (8.3)	85.2 (10.1)	80.2 (8.4; 53-112)
At 25 weeks	80.7 (8.3)	89.1 (11.0)	80.9 (8.5; 51-133)		81.0 (8.2)	86.8 (11.1)	81.2 (8.4; 53-133)
At 28 weeks	81.2 (8.4)	90.8 (12.3)	81.4 (8.6; 37-160)		81.5 (8.3)	90.8 (11.0)	81.7 (8.5; 53-133)
At 31 weeks	81.9 (8.5)	92.1 (12.1)	82.1 (8.7; 46-150)		82.3 (8.4)	93.5 (13.3)	82.6 (8.7; 54-134)
At 34 weeks	82.8 (8.9)	95.5 (13.3)	83.0 (9.2; 40-157)		83.1 (8.7)	96.9 (14.2)	83.5 (9.2; 55-137)
At 36 weeks	84.0 (9.3)	100.7 (13.3)	84.3 (9.7; 47-157)		84.4 (8.9)	100.3 (13.5)	84.8 (9.3; 57-148)
Preterm (%):							
No	95.2	72.6	94.6		95.0	73.9	94.4
Yes	4.8	27.4	5.4		5.0	26.1	5.6
Small for gestational age (%):							
No	90.5	74.2	90.1		90.4	76.1	90.0
Yes	9.5	25.8	9.9		9.6	23.9	10.0
Mean (SD; range for overall mean) maternal BMI	22.9 (3.8)	24.8 (5.2)	23.0 (3.8; 12-55)		24.9 (4.7)	26.1 (5.2)	24.9 (4.7; 14-50)
Maternal BMI categories (%):							
Underweight	5.9	3.5	5.9		2.7	3.1	2.7
Normal	72.7	60.6	72.4		58.0	44.0	57.6
Overweight	16.3	21.1	16.4		26.4	32.9	26.6
Obese	5.1	14.9	5.4		12.9	19.9	13.1
Mean (SD; range for overall mean) maternal height	163.9 (6.7)	163.0 (6.6)	163.9 (6.7; 124-201)		163.2 (6.4)	163.6 (6.4)	163.2 (6.4; 139-190)
Mean (SD; range for overall mean) maternal age (years)	27.9 (5.0)	27.5 (5.6)	27.9 (5.0; 15-44)		30.7 (3.8)	30.0 (3.9)	30.6 (3.8; 21-42)
Maternal age (%):							
≤34	90.3	86.8	90.2		86.1	89.8	86.2
>35	9.7	13.2	9.8		13.9	10.2	13.8
Parity (%):							
Nulliparous	45.3	70.1	45.9		49.7	85.2	50.7
1	34.8	17.8	34.4		33.7	9.1	33.0
≥2	19.9	12.1	19.7		16.7	5.7	16.3
Maternal smoking in pregnancy (%):							
No	73.9	84.2	74.1		83.3	86.5	83.4
Yes	26.1	15.8	25.9		16.7	13.5	16.6
Essential hypertension (%):							
No	96.5	88.4	96.3		97.6	88.5	97.4
Yes	3.5	11.6	3.7		2.4	11.5	2.6
Previous gestational hypertension (%):							
No	91.2	84.7	91.0		63.7	18.4	62.4
Yes	8.8	15.3	9.0		36.3	81.6	37.6
Diabetes (%):							
No	99.7	96.1	99.6		99.5	96.8	99.4
Yes	0.3	3.9	0.4		0.5	3.2	0.6
Previous gestational diabetes (%):							
No	99.5	98.8	99.5		92.0	78.8	91.7
Yes	0.5	1.2	0.5		8.0	21.2	8.3
Maternal ethnicity (%):							
White	97.3	95.8	97.2		95.4	97.7	95.4
Non-white	2.7	4.2	2.8		4.6	2.3	4.6

Table F in appendix 1 shows the unadjusted associations of each of the predictor variables with each of the outcomes.

### Prediction of pre-eclampsia

Table 2 shows the area under the receiver operating characteristic curves for prediction of pre-eclampsia for the model with only early pregnancy characteristics and subsequent models adding mean arterial pressure at different gestational ages from 20 to 36 weeks. The prediction model that included only early pregnancy maternal characteristics and first mean arterial pressure from the antenatal clinic had an area under the curve of around 0.77 in the development cohort (ALSPAC; model 1). This improved to 0.79 with addition of mean arterial pressure (model 2) at 20 weeks of gestation and became greater with increasing gestational age at which mean arterial pressure was measured, being 0.88 at 36 weeks of gestation. In the validation cohort (SWS) areas under the curve were similar to those in the development cohort, but the earliest gestational age at which there was evidence of an improvement in the area under the curve for model 2 compared with model 1 was at 28 weeks. We therefore focused on this gestational age when presenting calibration and predictive statistics. Models 3 and 4 performed similarly to model 2 at all gestational ages in ALSPAC and SWS (table G in appendix 1). Figure A in appendix 3 shows the ROC curves for all four models with mean arterial pressure measured at 28 weeks in ALSPAC and SWS.

**Table 2 tbl2:** Prediction models for pre-eclampsia at different gestational ages in Avon Longitudinal Study of Parents and Children (ALSPAC) and Southampton Women’s Survey (SWS) using imputed datasets. Figures are area under receiver operating characteristic curve (95% confidence intervals)

Gestational age of prediction (weeks)	Development cohort (ALSPAC)				Validation cohort (SWS)		
	Model 1*	Model 2†	P value for 2 *v* 1		Model 1*	Model 2†	P value for 2 *v* 1
20	0.77 (0.75 to 0.80)	0.79 (0.77 to 0.82)	0.002		0.79 (0.73 to 0.85)	0.80 (0.74 to 0.85)	0.47
25	0.77 (0.75 to 0.80)	0.80 (0.77 to 0.82)	<0.001		0.79 (0.73 to 0.85)	0.80 (0.75 to 0.86)	0.39
28	0.77 (0.75 to 0.80)	0.81 (0.79 to 0.84)	<0.001		0.79 (0.73 to 0.85)	0.84 (0.79 to 0.88)	0.003
31	0.77 (0.74 to 0.79)	0.82 (0.80 to 0.85)	<0.001		0.79 (0.73 to 0.85)	0.84 (0.79 to 0.89)	0.008
34	0.77 (0.74 to 0.80)	0.84 (0.82 to 0.87)	<0.001		0.81 (0.75 to 0.87)	0.86 (0.81 to 0.90)	0.01
36	0.77 (0.74 to 0.80)	0.88 (0.86 to 0.90)	<0.001		0.80 (0.74 to 0.87)	0.88 (0.84 to 0.93)	<0.001

*Includes maternal covariates and mean arterial pressure at first visit.

†As model 1 plus observed value of mean arterial pressure for gestational age.

Table H in appendix 1 and figure 1 show the calibration of model 2 at 28 weeks’ gestation for prediction of pre-eclampsia at 10ths of the risk score in the validation cohort (SWS). The model tended to overestimate the risk of pre-eclampsia, especially at higher values of the risk score, suggesting that we needed to recalibrate the model. Recalibration details are given in the appendix 2. After recalibration the risk of pre-eclampsia was well estimated by the model in the validation cohort (table H in appendix 1; fig 1).

**Figure fig1:**
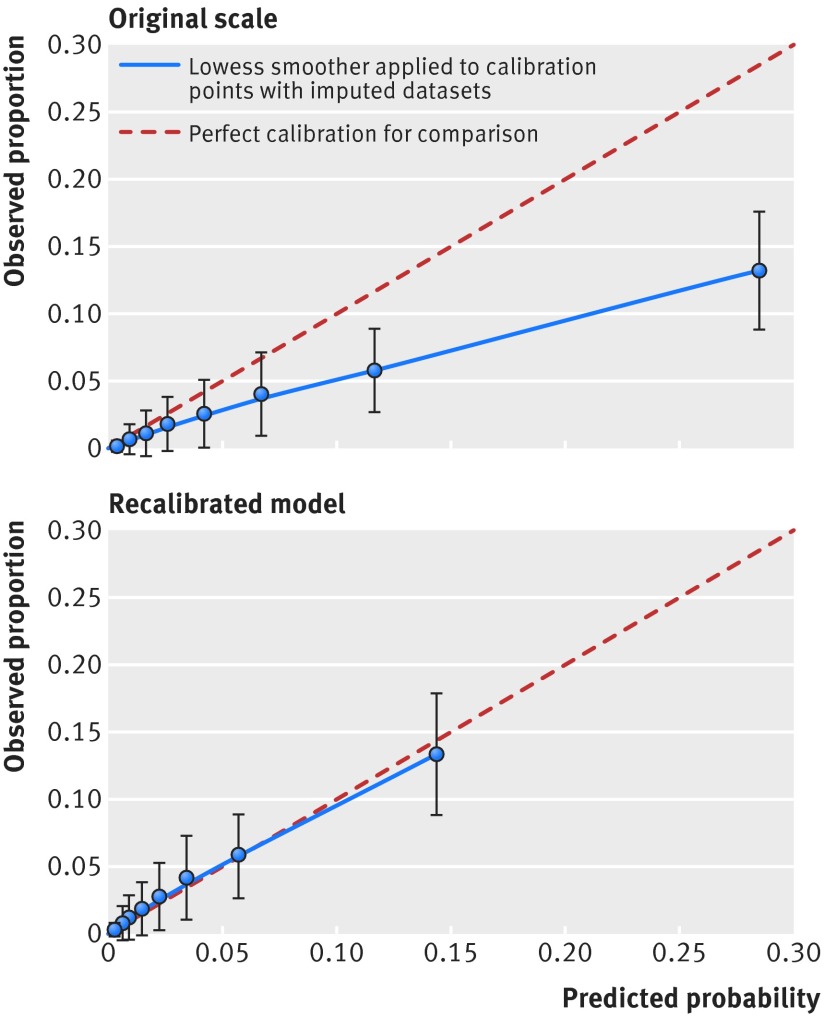
**Fig 1** Observed proportion of women who developed pre-eclampsia and 95% confidence interval in 10ths of predicted risk from model 2 at 28 weeks on original scale and with recalibrated model in Southampton Women’s Survey (n=3005) with imputed datasets. Model 2 includes maternal covariates and mean arterial pressure at first visit and at 28 weeks

Table 3 shows specificity, positive and negative predictive values, and positive and negative likelihood ratios at fixed sensitivities of 95% and 99% (meaning that 95% and 99% of women who developed preeclampsia would test positive) in ALSPAC and SWS for model 2 at 28 weeks. At these high sensitivities, as expected, there was a high false positive rate (low specificity), though we also observed a high negative predictive value (about 1) and low negative likelihood ratio, suggesting a strong ability to rule out pre-eclampsia in women who tested negative at these values of the risk score. Figure 2 shows the expected results of a sequential screening strategy to rule out pre-eclampsia, testing with model 1 at booking and then with model 2 from 20 weeks onwards, with the threshold of the risk score set according to a sensitivity of 99% at each gestational age in ALSPAC, and the threshold applied in SWS. Women are moved into the low risk group at any gestational age at which they test negative for pre-eclampsia, and once in the low risk group women are not included in subsequent screens. Based on this approach it is expected that at 31 weeks, 27 out of 28 women who had not yet delivered and would go on to develop pre-eclampsia would be included in the high risk group and that just over a third of all of the women would have been allocated to the low risk group with a reduced care schedule. Figure B in appendix 3 shows the equivalent result with the threshold of the risk score that gave a 95% sensitivity in ALSPAC.

**Table 3 tbl3:** Prediction of pre-eclampsia in Avon Longitudinal Study of Parents and Children (ALSPAC) and Southampton Women’s Survey (SWS) with model 2* at 28 weeks’ gestation for fixed sensitivities using imputed datasets

Sensitivity	Risk score threshold	Specificity (95% CI)	Positive predictive value (95% CI)	Negative predictive value (95% CI)	Positive likelihood ratio (95% CI)	Negative likelihood ratio (95% CI)	No (%) testing positive	No (%) true positive	No (%) false positive	No (%) true negative	No (%) false negative
**ALSPAC**											
0.95	0.008	0.37 (0.31 to 0.42)	0.04 (0.03 to 0.04)	1.00 (0.99 to 1.00)	1.50 (1.37 to 1.64)	0.14 (0.08 to 0.23)	8316 (64.1)	297 (2.3)	8019 (61.9)	4633 (35.7)	16 (0.1)
0.99	0.003	0.15 (0.13 to 0.17)	0.03 (0.02 to 0.03)	1.00 (1.00 to 1.00)	1.16 (1.13 to 1.20)	0.07 (0.02 to 0.21)	11068 (85.4)	310 (2.4)	10758 (83.0)	1894 (14.6)	3 (0.0)
**SWS (without recalibration, using ALSPAC risk score thresholds)**											
1.00 (0.98 to 1.00)	0.008	0.31 (0.29 to 0.33)	0.04 (0.03 to 0.05)	1.00 (1.00 to 1.00)	1.44† (1.40 to 1.50)	0.02^†^ (0.00 to 0.32)	2086 (69.6)	87 (2.9)	2000 (66.7)	913 (30.4)	0 (0.0)
1.00 (1.00 to 1.00)	0.003	0.10 (0.09 to 0.11)	0.03 (0.03 to 0.04)	1.00 (1.00 to 1.00)	1.11† (1.09 to 1.13)	0.06^†^ (0.00 to 0.90)	2699 (90.0)	87 (2.9)	2612 (87.1)	300 (10.0)	0 (0.0)
**SWS (after recalibration)**											
0.95	0.011	0.47 (0.40 to 0.55)	0.05 (0.04 to 0.06)	1.00 (0.99 to 1.00)	1.82 (1.56 to 2.11)	0.10 (0.04 to 0.26)	1617 (53.9)	83 (2.8)	1534 (51.2)	1379 (46.0)	4 (0.1)
0.99	0.006	0.33 (0.22 to 0.44)	0.04 (0.03 to 0.05)	1.00 (1.00 to 1.00)	1.49 (1.28 to 1.73)	0.04 (0.00 to 0.27)	2037 (67.9)	86 (2.9)	1951 (65.1)	961 (32.0)	1 (0.0)

*Includes maternal covariates, mean arterial pressure at first visit, and observed value of mean arterial pressure at 28 weeks.

†Continuity correction applied in calculation of likelihood ratios in multiply imputed datasets where 2×2 contingency tables of test *v* disease status had zero cells. Constant of 0.5 has been added to all cells in these cases.

**Figure fig2:**
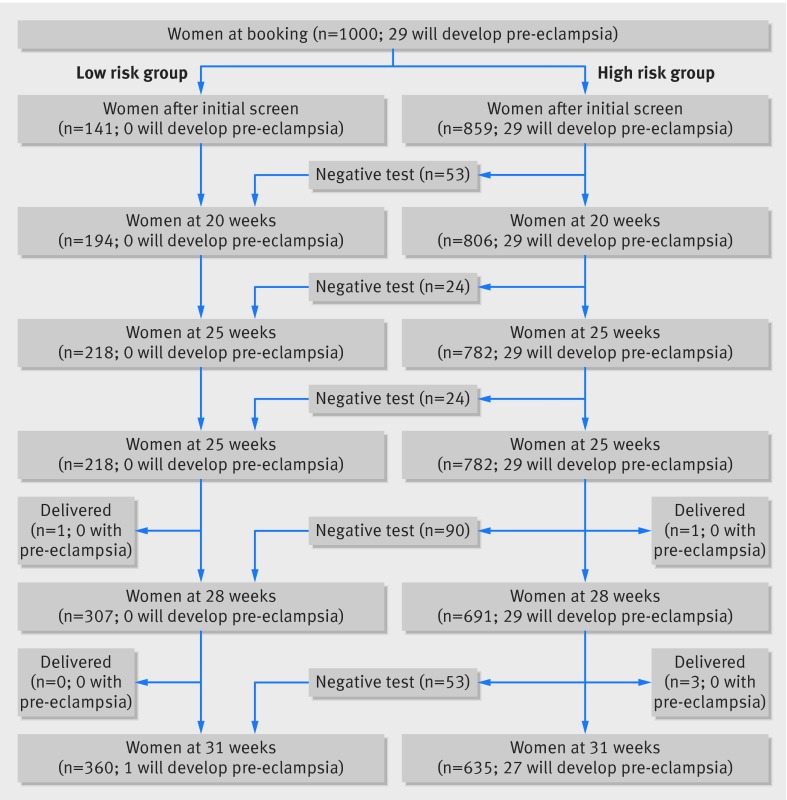
**Fig 2** Expected numbers of women allocated to low and high risk groups for pre-eclampsia from cohort of 1000 women based on testing sequentially with model 1 at booking followed by model 2 at subsequent visits with threshold of 0.0047 at each gestational age up to 31 weeks. After initial screen, only high risk group are included in subsequent screening tests. To obtain these numbers we determined average threshold giving 99% sensitivity at each stage in ALSPAC (including high risk group only after initial screen) and applied this threshold in SWS. Model 1 includes maternal covariates and mean arterial pressure at first visit; model 2 additionally includes mean arterial pressure at gestational age of testing

Table I in appendix 1 shows predictive statistics fixing low false positive rates of 25%, 10%, and 5% (high specificities) for model 2 at 28 weeks. For a 5% false positive rate the sensitivity was 35% in ALSPAC and 32% in SWS, suggesting that the model had a weaker ability to rule in pre-eclampsia than to rule it out.

#### Prediction of preterm birth

In both ALSPAC and the SWS, the model including only maternal characteristics in early pregnancy and mean arterial pressure at the initial visit had an area under the curve of 0.61 for prediction of preterm birth (model 1; table J in appendix 1). There was little improvement from including the current mean arterial pressure (model 2) or the deviation of mean arterial pressure from the normogram (model 3) until 31 weeks’ gestation, when the area under the curve rose to 0.63 in ALSPAC for models 2 and 3. Similar areas under the curve were seen in the SWS, except that an improvement was not seen until 34 weeks for models 2 and 3 compared with model 1.

If we excluded cases of pre-eclampsia from our analysis, areas under the curve for prediction of preterm birth were generally slightly lower in both cohorts, but there remained an improvement from the addition of mean arterial pressure measurements from 31 weeks onwards in ALSPAC and at 34 weeks in SWS (table K in appendix 1).

#### Prediction of babies small for gestational age

In ALSPAC and SWS, the model including only maternal characteristics and mean arterial pressure at the initial visit had an are under the curve of around 0.70 for the prediction of small for gestational age (model 1; table L in appendix 1). Inclusion of either the current mean arterial pressure at any later gestational age (model 2) or deviation of mean arterial pressure from the normogram (model 3) or both (model 4) did not improve this in ALSPAC or SWS.

## Discussion

### Principal findings

We have developed and compared prediction models for pre-eclampsia and two of its associated adverse outcomes—preterm birth and small for gestational age—using observed antenatal blood pressure measurements (with diastolic and systolic entered into our models jointly as mean arterial pressure). We did this at varying gestational ages during pregnancy and externally validated these models in a similar large population based prospective pregnancy cohort. From 28 weeks’ gestation onwards, the use of the current blood pressure in prediction models improved the identification of women who would develop pre-eclampsia compared with using maternal characteristics measured in early pregnancy including the first measurement; this could be used to facilitate stratified care. We found, however, that comparison against normograms allowing for distinctly different expected trajectories of blood pressure across pregnancy for different subgroups of women based on pre-pregnancy BMI, parity, smoking, and initial blood pressure did not improve the discriminative ability of the prediction models compared with using the observed measurement alone. Measures were of little value in predicting preterm birth until around 31 weeks’ gestation and did not aid in the prediction of small for gestational age at any gestational age after 20 weeks.

### Study strengths and limitations

We used two large prospective cohort studies, one to develop the prediction models and the second to externally validate them. The detailed measurements of blood pressure in each of the studies allowed us to evaluate the predictive ability of these measurements at multiple gestational ages across pregnancy. We also used multiple imputation to maximise the sample size available. The ALSPAC pregnancies occurred over 20 years ago, and, as then, there might have been changes in clinical practice. The models, however, were validated in the SWS, where the pregnancies occurred more recently (8-16 years ago). Several of the predictor variables used were self reported so could be prone to measurement error. This is likely to reflect the way that information is obtained in clinical practice. We needed to recalibrate the prediction model for pre-eclampsia in the SWS. This is probably because of different definitions of pre-eclampsia in the two cohorts; in ALSPAC pre-eclampsia was defined by applying a standard definition to all measurements of blood pressure and proteinuria in pregnancy whereas in SWS a record of a clinical diagnosis of pre-eclampsia during pregnancy was used. This suggests that the risk score requires recalibration according to different definitions of pre-eclampsia. The areas under the curve, however, were similar in the SWS to those in ALSPAC suggesting that the predictive ability of measurements of blood pressure in the second half of pregnancy is not dependent on the use of a particular definition of pre-eclampsia. The number of cases of pre-eclampsia in the SWS (n=88) was slightly lower than the recommended 100 events for validation,^35^ and our study might therefore have been slightly underpowered to detect differences in model fit. Despite this, our confidence intervals were of a reasonable width, and we were able to detect both the need for recalibration in SWS and an improvement in the area under the curve for pre-eclampsia from the addition of blood pressure measures from 28 weeks onwards in this cohort. We could not distinguish between medically indicated and spontaneous preterm births in our analyses, but our sensitivity analysis excluding women who had pre-eclampsia is likely to exclude most medically indicated preterm births related to high blood pressure.

### Comparisons with other studies

The area under the curve for our prediction model for pre-eclampsia using mean arterial pressure at 28 weeks was 0.81 in ALSPAC and 0.83 in SWS. This is higher than the area found in a model based on maternal clinical risk factors (including mean arterial pressure) in nulliparous women only in the SCOPE study at 15 weeks’ gestation (0.76 in training dataset and 0.71 after validation).^15^ Table M in appendix 1 shows the risk factors included in that model (not all of these were available in ALSPAC and SWS for us to compare our model directly with the SCOPE model). A recent systematic review identified 69 prediction models for pre-eclampsia but reported that only five of these had been externally validated.^36^ The validated models all included uterine artery Doppler measures or biomarkers that require laboratory testing that is not currently part of routine practice and is too expensive for use in low or middle income countries.^21^ ^37^ ^38^ ^39^ ^40^ Our prediction model uses readily available measures and allows for a sequential screening approach at each antenatal appointment that could be used in conjunction with screening in early pregnancy to update each woman’s risk estimate as pregnancy progresses. In a meta-analysis, mean arterial pressure in the second trimester was found to predict pre-eclampsia well, with an are under the curve of 0.76 in low risk populations.^41^ We have shown that this increases with gestation and that mean arterial pressure in the second half of pregnancy contributes additional predictive ability compared with using early pregnancy clinical characteristics alone.

### Conclusions and policy implications

Current models of antenatal care focus on a series of repeat scheduled antenatal visits with recurrent screening for pre-eclampsia. In the UK, nulliparous women will have 12 healthcare assessments across gestation with nine of these visits occurring after 20 weeks’ gestation, while multiparous women will have nine assessments with six occurring after 20 weeks, missing appointments at 25, 31, and 40 weeks.^29^ Similar stratified (based on parity) care occurs in other Western countries. A potential use of our model is for nulliparous women who are screened as low risk to be transferred to a reduced schedule similar to that of a multiparous woman. Conversely, it might be appropriate to transfer multiparous women screening as high risk to the more intensive schedule of a nulliparous woman. We have provided two examples of how care schedules could be altered in this way following sequential screening (fig 3). The decision of whether to increase the schedule of antenatal appointments for women testing as high risk versus decreasing the schedule for women classified as low risk, however, will depend on the setting and resources available for screening. A systematic review of randomised controlled trials that compared standard antenatal care with reduced care schedules found no difference in the rates of detection of pre-eclampsia in studies of low risk women in high income countries.^42^ Nevertheless, in low income countries the standard schedule of antenatal care might consist of only four visits across pregnancy, and a low proportion of women achieve full attendance at these; here a focus on increasing the number of visits or increasing the awareness of the importance of these visits for women who screen as high risk might be most beneficial.^43^ It is also important to note that we have not taken into account reasons for monitoring pregnant women other than pre-eclampsia, and these must also be considered when this care schedule is planned.

**Figure fig3:**
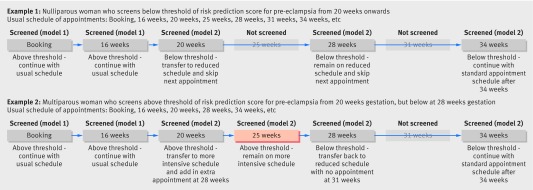
**Fig 3** Examples of possible alterations to scheduled appointment regimens based on sequential screening

Further qualitative research is required to evaluate the views of clinicians and pregnant women on the frequency of antenatal care as many women find this regular contact with healthcare professionals reassuring during pregnancy.^42^ This would inform the potential benefits and harms of different screening strategies and could be followed up with decision curve analysis to weigh these against each other.^44^ If the prediction models were to be used widely this would require the development of a tool for clinical use. Such a tool could be incorporated into a smart phone app, and future research could focus on evaluating the use of this tool in low and middle income settings, in a similar way to the development and evaluation of tools for smart phones that has occurred in ophthalmology.^45^ ^46^ Finally, extension of the models with additional measures such as the number and gestation of previous preterm deliveries or cervical length for preterm birth^47^ or first trimester serum concentrations of pregnancy proteins for small for gestational age,^48^ ^49^ given that placental dysfunction is likely to underlie growth restriction, might be able to improve prediction of the adverse outcomes associated with pre-eclampsia.

In conclusion, measurements of blood pressure recorded during the second half of pregnancy, used in conjunction with blood pressure early in pregnancy and other maternal risk factors, can improve the identification of women who are at risk of developing pre-eclampsia later in pregnancy and could be used to differentiate women who require more intensive monitoring from those who are likely to have a normal pregnancy. As there is little value in additionally using these later pregnancy measurements to predict preterm birth and small for gestational age, however, disease specific models will be required to facilitate stratified care for the range of pregnancy complications.

**What is already known on this topic**

Blood pressure measured at the booking appointment for antenatal care is often used in combination with other clinical risk factors in prediction models to identify women at risk of developing pre-eclampsia later in pregnancy
Studies that have compared patterns of change in blood pressure across pregnancy have shown that, as well as having a higher initial blood pressure, women who develop pre-eclampsia have a steeper rise in blood pressure in the second half of pregnancy

**What this study adds**

The addition of routinely collected measurements of blood pressure taken in the second half of pregnancy to prediction models for pre-eclampsia improves their performance compared with using early risk factors alone
Use of these prediction models sequentially at each antenatal appointment to rule out women who are at low risk of developing pre-eclampsia could enable these women to be transferred to a reduced antenatal care schedule
Blood pressure measurements in the second half of pregnancy are of little benefit in the prediction of preterm birth or small for gestational age offspring

## Notes

We thank all of the families who took part in this study, the midwives for their help in recruiting them, and the whole ALSPAC team, which includes interviewers, computer and laboratory technicians, clerical workers, research scientists, volunteers, managers, receptionists, and nurses. We also thank the parents and children in the SWS who participated and the SWS staff who collected and processed the data.

Up to August 2016 the corresponding author is D Lawlor d.a.lawlor@bristol.ac.uk

Contributors: CM-W, RJS, BLDS, DAL, and KT contributed to the conception and design of the study and planned the statistical analysis. HI prepared the SWS dataset and HI and SC advised on matters relating to this cohort. CM-W completed the analyses and wrote the first draft of the paper. All authors contributed to the interpretation of findings, revising the manuscript for important intellectual content, and approved the final version to be published. All authors had full access to all of the data (including statistical reports and tables) in the study and can take responsibility for the integrity of the data and the accuracy of the data analysis. CM-W is guarantor.

Ethical approval: The study was approved by the ALSPAC law and ethics committee and the NHS local ethics committee.

Transparency declaration: The lead author (the manuscript’s guarantor) affirms that this manuscript is an honest, accurate, and transparent account of the study being reported; that no important aspects of the study have been omitted; and that any discrepancies from the study as planned have been explained.

Data sharing: No additional data available; collaborators can access existing data in either ALSPAC (www.bristol.ac.uk/alspac) or SWS (www.mrc.soton.ac.uk/sws) through their respective websites which provide information on how to do this.

## Notes

Cite this as: *BMJ* 2015;351:h5948

## References

[1] Centre for Maternal and Child Enquiries (CMACE). Saving mothers’ lives: reviewing maternal deaths to make motherhood safer: 2006-08. The Eighth Report on Confidential Enquiries into Maternal Deaths in the United Kingdom. Br J Obstet Gynaecol 2011;118(suppl 1):1-203. 10.1111/j.1471-0528.2010.02847.x 21356004

[2] Centre for Maternal and Child Enquiries (CMACE). Perinatal mortality 2009: United Kingdom. CMACE, 2011.

[3] Ananth CV, Peedicayil A, Savitz DA. Effect of hypertensive diseases in pregnancy on birth-weight, gestational duration, and small-for-gestational-age births. Epidemiology 1995;6:391-95. 7548347 10.1097/00001648-199507000-00011

[4] Bakker R, Steegers EAP, Hofman A, Jaddoe VWV. Blood pressure in different gestational trimesters, fetal growth, and the risk of adverse birth outcomes. The Generation R Study. Am J Epidemiol 2011;174:797-806. 21859836 10.1093/aje/kwr151

[5] Xiong X, Demianczuk NN, Saunders LD, Wang FL, Fraser WD. Impact of preeclampsia and gestational hypertension on birth weight by gestational age. Am J Epidemiol 2002;155:203-9. 11821244 10.1093/aje/155.3.203

[6] Brown MA, Lindheimer MD, de Swiet M, Van Assche A, Moutquin JM. The classification and diagnosis of the hypertensive disorders of pregnancy: statement from the International Society for the Study of Hypertension in Pregnancy (ISSHP). Hypertens Pregnancy 2001;20:IX-XIV. 12044323 10.1081/PRG-100104165

[7] Greer IA. Pre-eclampsia matters. BMJ 2005;330:549-50. 15760971 10.1136/bmj.330.7491.549 PMC554013

[8] Milne F, Redman C, Walker J, Baker P, Bradley J, Cooper C, et al. The pre-eclampsia community guideline (PRECOG): how to screen for and detect onset of pre-eclampsia in the community. BMJ 2005;330:576-80. 15760998 10.1136/bmj.330.7491.576 PMC554032

[9] Steegers EA, von DP, Duvekot JJ, Pijnenborg R. Pre-eclampsia. Lancet 2010;376:631-44. 20598363 10.1016/S0140-6736(10)60279-6

[10] Koopmans CM, Bijlenga D, Groen H, et al. Induction of labour versus expectant monitoring for gestational hypertension or mild pre-eclampsia after 36 weeks’ gestation (HYPITAT): a multicentre, open-label randomised controlled trial. Lancet 2009;374:979-88. 19656558 10.1016/S0140-6736(09)60736-4

[11] Macdonald-Wallis C, Lawlor DA, Fraser A, May M, Nelson SM, Tilling K. Blood pressure change in normotensive, gestational hypertensive, preeclamptic, and essential hypertensive pregnancies. Hypertension 2012;59:1241-48. 22526257 10.1161/HYPERTENSIONAHA.111.187039 PMC3378662

[12] Gaillard R, Bakker R, Willemsen SP, Hofman A, Steegers EA, Jaddoe VW. Blood pressure tracking during pregnancy and the risk of gestational hypertensive disorders: the Generation R Study. Eur Heart J 2011;32:3088-97. 21821845 10.1093/eurheartj/ehr275

[13] Steer PJ, Little MP, Kold-Jensen T, Chapple J, Elliott P. Maternal blood pressure in pregnancy, birth weight, and perinatal mortality in first births: prospective study. BMJ 2004;329:1312-14. 15561733 10.1136/bmj.38258.566262.7C PMC534837

[14] Macdonald-Wallis C, Tilling K, Fraser A, Nelson SM, Lawlor DA. Associations of blood pressure change in pregnancy with fetal growth and gestational age at delivery: findings from a prospective cohort. Hypertension 2014;64:36-44. 24821945 10.1161/HYPERTENSIONAHA.113.02766 PMC4150069

[15] North RA, Mccowan LME, Dekker GA, et al. Clinical risk prediction for pre-eclampsia in nulliparous women: development of model in international prospective cohort. BMJ 2011;342:d1875. 21474517 10.1136/bmj.d1875 PMC3072235

[16] Akolekar R, Syngelaki A, Sarquis R, Zvanca M, Nicolaides KH. Prediction of early, intermediate and late pre-eclampsia from maternal factors, biophysical and biochemical markers at 11-13 weeks. Prenat Diagn 2011;31:66-74. 21210481 10.1002/pd.2660

[17] Broughton Pipkin F, Sharif J, Lal S. Predicting high blood pressure in pregnancy: a multivariate approach. J Hypertens 1998;16:221-9. 9535150 10.1097/00004872-199816020-00013

[18] Myatt L, Clifton RG, Roberts JM, et al. First-trimester prediction of preeclampsia in nulliparous women at low risk. Obstet Gynecol 2012;119:1234-42. 22617589 10.1097/AOG.0b013e3182571669 PMC3360523

[19] Nijdam ME, Janssen KJM, Moons KGM, et al. Prediction model for hypertension in pregnancy in nulliparous women using information obtained at the first antenatal visit. J Hypertens 2010;28:119-26. 19907344 10.1097/HJH.0b013e32833191b0

[20] Poon LCY, Kametas NA, Pandeva I, Valencia C, Nicolaides KH. Mean arterial pressure at 11(+0) to 13(+6) weeks in the prediction of preeclampsia. Hypertension 2008;51:1027-33. 18259003 10.1161/HYPERTENSIONAHA.107.104646

[21] Poon LCY, Kametas NA, Maiz N, Akolekar R, Nicolaides KH. First-trimester prediction of hypertensive disorders in pregnancy. Hypertension 2009;53:812-18. 19273739 10.1161/HYPERTENSIONAHA.108.127977

[22] Kenny LC, Black MA, Poston L, et al. Early pregnancy prediction of preeclampsia in nulliparous women, combining clinical risk and biomarkers: the screening for pregnancy endpoints (SCOPE) international cohort study. Hypertension 2014;64:644-52. 25122928 10.1161/HYPERTENSIONAHA.114.03578

[23] Chappell LC, Seed PT, Myers J, et al. Exploration and confirmation of factors associated with uncomplicated pregnancy in nulliparous women: prospective cohort study. BMJ 2013;347:f6398. 24270055 10.1136/bmj.f6398 PMC3898573

[24] Macdonald-Wallis C, Silverwood R, Fraser A, et al. Gestational-age-specific reference ranges for blood pressure in pregnancy: findings from a prospective cohort. J Hypertens 2014;33:10. 10.1097/HJH.0000000000000368 PMC4255761 25255393

[25] Collins GS, Reitsma JB, Altman DG, Moons KG. Transparent reporting of a multivariable prediction model for individual prognosis or diagnosis (TRIPOD): the TRIPOD statement. BMJ 2015;350:g7594. 25569120 10.1136/bmj.g7594

[26] Fraser A, Macdonald-Wallis C, Tilling K, et al. Cohort profile: the Avon Longitudinal Study of Parents and Children: ALSPAC mothers cohort. Int J Epidemiol 2012;42:14. 10.1093/ije/dys066 PMC3600619 22507742

[27] Steyerberg EW, Moons KG, van der Windt DA, et al. Prognosis research strategy (PROGRESS) 3: prognostic model research. PLoS Med 2013;10:e1001381. 23393430 10.1371/journal.pmed.1001381 PMC3564751

[28] Inskip HM, Godfrey KM, Robinson SM, et al. Cohort profile: the Southampton Women’s Survey. Int J Epidemiol 2006;35:42-8. 16195252 10.1093/ije/dyi202 PMC4579566

[29] National Institute for Health and Clinical Excellence. Antentatal care. NICE Clinical Guideline CG62. NICE, 2008. http://guidance.nice.org.uk/CG62.

[30] Rubin DB. Multiple imputation after 18+ years. J Am Stat Assoc 1996;91:473-89.

[31] Vergouwe Y, Royston P, Moons KGM, Altman DG. Development and validation of a prediction model with missing predictor data: a practical approach. J Clin Epidemiol 2010;63:205-14. 19596181 10.1016/j.jclinepi.2009.03.017

[32] Sterne JA, White IR, Carlin JB, et al. Multiple imputation for missing data in epidemiological and clinical research: potential and pitfalls. BMJ 2009;338:b2393. 19564179 10.1136/bmj.b2393 PMC2714692

[33] Steyerberg EW, Borsboom GJ, van Houwelingen HC, Eijkemans MJ, Habbema JD. Validation and updating of predictive logistic regression models: a study on sample size and shrinkage. Stat Med 2004;23:2567-86. 15287085 10.1002/sim.1844

[34] Leckie G, Charlton C. runmlwin: Stata module for fitting multilevel models in the MLwiN software package. Centre for Multilevel Modelling, University of Bristol, 2011.

[35] Vergouwe Y, Steyerberg EW, Eijkemans MJC, Habbema JDF. Substantial effective sample sizes were required for external validation studies of predictive logistic regression models. J Clin Epidemiol 2005;5:475-83. 10.1016/j.jclinepi.2004.06.017 15845334

[36] Kleinrouweler CE, Cheong-See FM, Collins GS, et al. Prognostic models in obstetrics: available, but far from applicable. Am J Obstet Gynecol 2015, 10.1016/j.ajog.2015.06.013. 10.1016/j.ajog.2015.06.013 26070707

[37] Poon LC, Maiz N, Valencia C, Plasencia W, Nicolaides KH. First-trimester maternal serum pregnancy-associated plasma protein-A and pre-eclampsia. Ultrasound Obst Gynecol 2009;33:23-33. 19090499 10.1002/uog.6280

[38] Onwudiwe N, Yu CK, Poon LC, Spiliopoulos I, Nicolaides KH. Prediction of pre-eclampsia by a combination of maternal history, uterine artery Doppler and mean arterial pressure. Ultrasound Obstet Gynecol 2008;32:877-83. 18991324 10.1002/uog.6124

[39] Plasencia W, Maiz N, Bonino S, Kaihura C, Nicolaides KH. Uterine artery Doppler at 11 + 0 to 13 + 6 weeks in the prediction of pre-eclampsia. Ultrasound Obstet Gynecol 2007;30:742-9. 17899573 10.1002/uog.5157

[40] Plasencia W, Maiz N, Poon L, Yu C, Nicolaides KH. Uterine artery Doppler at 11 + 0 to 13 + 6 weeks and 21 + 0 to 24 + 6 weeks in the prediction of pre-eclampsia. Ultrasound Obstet Gynecol 2008;32:138-46. 18634131 10.1002/uog.5402

[41] Cnossen JS, Vollebregt KC, de Vrieze N, et al. Accuracy of mean arterial pressure and blood pressure measurements in predicting pre-eclampsia: systematic review and meta-analysis. BMJ 2008;336:1117-20. 18480117 10.1136/bmj.39540.522049.BE PMC2386627

[42] Carroli G, Villar J, Piaggio G, et al. WHO systematic review of randomised controlled trials of routine antenatal care. Lancet 2001;357:1565-70. 11377643 10.1016/S0140-6736(00)04723-1

[43] Pell C, Menaca A, Were F, et al. Factors affecting antenatal care attendance: results from qualitative studies in Ghana, Kenya and Malawi. PLoS One 2013;8:e53747. 23335973 10.1371/journal.pone.0053747 PMC3546008

[44] Vickers AJ, Elkin EB. Decision curve analysis: a novel method for evaluating prediction models. Med Decis Making 2006;26:565-74. 17099194 10.1177/0272989X06295361 PMC2577036

[45] Bastawrous A, Cheeseman RC, Kumar A. iPhones for eye surgeons. Eye 2012;26:343-54. 22399022 10.1038/eye.2012.6 PMC3298974

[46] Giardini ME, Livingstone IA, Jordan S, et al. A smartphone based ophthalmoscope. Proceedings of Annual International Conference of the IEEE Engineering in Medicine and Biology Society. IEEE Engineering in Medicine and Biology Society. 2014;2014:2177-80. 10.1109/EMBC.2014.6944049 25570417

[47] Heath VCF, Southall TR, Souka AP, Elisseou A, Nicolaides KH. Cervical length at 23 weeks of gestation: prediction of spontaneous preterm delivery. Ultrasound Obst Gynecol 1998;12:312-17. 9819868 10.1046/j.1469-0705.1998.12050312.x

[48] Smith GC, Stenhouse EJ, Crossley JA, Aitken DA, Cameron AD, Connor JM. Early pregnancy levels of pregnancy-associated plasma protein a and the risk of intrauterine growth restriction, premature birth, preeclampsia, and stillbirth. J Clin Endocrinol Metab 2002;87:1762-7. 11932314 10.1210/jcem.87.4.8430

[49] Dugoff L, Hobbins JC, Malone FD, et al. First-trimester maternal serum PAPP-A and free-beta subunit human chorionic gonadotropin concentrations and nuchal translucency are associated with obstetric complications: a population-based screening study (the FASTER Trial). Am J Obstet Gynecol 2004;191:1446-51. 15507981 10.1016/j.ajog.2004.06.052

