# Supplementary material for: Antenatal blood pressure for prediction of pre-eclampsia, preterm birth, and small for gestational age babies: development and validation in two general population cohorts
Source: BMJ. 2015 Nov 17;351:h5948. doi: 10.1136/bmj.h5948 (PMC4647185; doi:10.1136/bmj.h5948)
Supplement: Supplementary file 1 [file macc026935.ww1_default.pdf]

Appendix 1 Supplementary tables A-M [posted as supplied by author]

Table A Comparison of the sources and definitions of outcomes and predictor variables in ALSPAC and SWS

Variable	How this was measured/defined in ALSPAC	How this was measured/defined in SWS
Preeclampsia	Derived from all blood pressure and urine dipstick measurements taken during pregnancy: SBP ≥ 140 mmHg and/or DBP ≥ 90 mmHg along with proteinuria of 1+ or more on 2 occasions after 20 weeks gestation in a previously normotensive woman.	Diagnosis of preeclampsia recorded in and abstracted from obstetric records.
Preterm birth	Gestational age at delivery was calculated based on the final clinical estimate of the expected delivery date from obstetric records (mainly based on the last menstrual period date but for a small (unknown) proportion updated following a dating scan).<br><br>Preterm birth was defined as delivery at < 37 weeks.	A computerised algorithm was used to derive gestational age using menstrual data (66%) or, when these were uncertain or discrepant with ultrasound assessments, fetal anthropometry in early pregnancy was used.<br><br>Preterm birth was defined as delivery at < 37 weeks.
Small for gestational age	Birth weight was internally standardised for gestational age at birth and small for gestational age was defined as below the 10th percentile of this measure.	Birth weight was internally standardised for gestational age at birth and small for gestational age was defined as below the 10th percentile of this measure.
Initial MAP	The first antenatal measurement of MAP (abstracted from obstetric records), provided that this occurred before 18 weeks gestation	The first antenatal measurement of MAP (abstracted from obstetric records), provided that this occurred before 18 weeks gestation
MAP at X weeks gestation	Derived from the closest measure of MAP in the obstetric records occurring within +/- 2 weeks of gestational age X	Derived from the closest measure of MAP in the obstetric records occurring within +/- 2 weeks of gestational age X
Maternal BMI	Derived from self-reported maternal height and pre-pregnancy weight from a questionnaire administered in pregnancy	Derived from height measured by research staff prior to pregnancy and self-reported pre-pregnancy weight from an interview at 11 weeks gestation.

Maternal height	Self-reported in a questionnaire administered in pregnancy.	Measured by research staff prior to pregnancy.
Maternal age	Abstracted from obstetric records.	Abstracted from obstetric records.
Maternal parity	Self-reported in a questionnaire administered in pregnancy.	Self-reported at an interview prior to pregnancy.
Maternal smoking in pregnancy	Self-reported in a questionnaire at 18 weeks gestation. Women who reported smoking either in the first 3 months of pregnancy or within 2 weeks of the 18 week questionnaire were classed as having smoked during pregnancy.	Self-reported at interviews at 11 and 34 weeks gestation. Women who reported smoking on either occasion were classed as having smoked during pregnancy. An algorithm incorporating information from prior to pregnancy and at 6 months postpartum was used to infer smoking status where one or both measures of smoking were missing.
Pre-existing maternal hypertension	Self-reported in a questionnaire administered in pregnancy as having previously had hypertension outside of pregnancy.	Self-reported at an interview at 11 weeks gestation as having previously had high blood pressure outside of pregnancy.
Previous gestational hypertension	Self-reported in a questionnaire administered in pregnancy as having previously had hypertension but only in pregnancy.	Self-reported at an interview at 11 weeks gestation as having previously had high blood pressure in pregnancy.
Pre-existing diabetes	Self-reported in a questionnaire administered in pregnancy as having previously had diabetes outside of pregnancy.	Self-reported at an interview at 11 weeks gestation as having previously had diabetes outside of pregnancy.
Previous gestational diabetes	Self-reported in a questionnaire administered in pregnancy as having previously had diabetes but only in pregnancy.	Self-reported at an interview at 11 weeks gestation as having previously had diabetes in pregnancy.
Maternal non-white ethnicity	Self-reported in a questionnaire administered during pregnancy. All ethnicities other than “white” were classed as “non-white”.	Self-reported at an interview prior to pregnancy. All ethnicities other than “white” were classed as “non-white”.
Offspring sex	Abstracted from obstetric records.	Abstracted from obstetric records.

Table B Summary of risk factors included in prediction models for preeclampsia, preterm birth and small for gestational age at each stage of gestation

**Model**	**Preeclampsia**	**Preterm birth**	**Small for gestational age**
Model 1	Initial MAP (continuous)<br>Maternal BMI (continuous)<br>Maternal height (continuous)<br>Maternal age over 35 years (binary)<br>Maternal parity (3 categories: 0 (ref), 1, 2+)<br>Maternal smoking in pregnancy (binary)<br>Pre-existing hypertension (binary)<br>Previous gestational hypertension (binary)<br>Pre-existing diabetes (binary)<br>Previous gestational diabetes (binary)<br>Maternal non-white ethnicity (binary	Initial MAP (continuous)<br>Maternal BMI (4 categories: underweight (ref), normal, overweight, obese)<br>Maternal height (continuous)<br>Maternal parity (3 categories: 0 (ref), 1, 2+)<br>Pre-existing hypertension (binary)<br>Previous gestational hypertension (binary)<br>Pre-existing diabetes (binary)<br>Previous gestational diabetes (binary)<br>Maternal non-white ethnicity (binary)	Initial MAP (continuous)<br>Maternal BMI (4 categories: underweight (ref), normal, overweight, obese)<br>Maternal height (continuous)<br>Maternal age over 35 years (binary)<br>Maternal parity (3 categories: 0 (ref), 1, 2+)<br>Maternal smoking in pregnancy (binary)<br>Pre-existing hypertension (binary)<br>Previous gestational hypertension (binary)<br>Previous gestational diabetes (binary)<br>Maternal non-white ethnicity (binary)
Model 2	As Model 1 plus current MAP (continuous)	As Model 1 plus current MAP (continuous)	As Model 1 plus current MAP (continuous)
Model 3	As Model 1 plus current deviation of MAP from the normative value (continuous)	As Model 1 plus current deviation of MAP from the normative value (continuous)	As Model 1 plus current deviation of MAP from the normative value (continuous)
Model 4	As Model 1 plus both current MAP and current deviation of MAP from the normative value (continuous)	As Model 1 plus both current MAP and current deviation of MAP from the normative value (continuous)	As Model 1 plus both current MAP and current deviation of MAP from the normative value (continuous)

Table C Cumulative number of women who had delivered and developed preeclampsia at each gestational age

**Gestational age**	**Number (%) of participants who had delivered**		**Number (%) of participants who had developed preeclampsia in ALSPAC**
	**ALSPAC**	**SWS**	
**20 weeks**	0 (0.0)	0 (0.0)	0 (0.0)
**25 weeks**	7 (0.1)	0 (0.0)	4 (0.0)
**28 weeks**	31 (0.2)	6 (0.2)	15 (0.1)
**31 weeks**	89 (0.7)	17 (0.6)	36 (0.3)
**34 weeks**	207 (1.6)	40 (1.3)	60 (0.5)
**36 weeks**	431 (3.3)	102 (3.4)	107 (0.8)

Table D Characteristics of pregnancies in the observed and imputed data in ALSPAC and the SWS

**Characteristics**		**ALSPAC**				**SWS**			
		N (%) missing	N with data	Observed Mean (SD)/ %	Imputed Mean (SD)/ %	N (%) missing	N with data	Observed Mean (SD)/ %	Imputed Mean (SD)/ %
Preeclampsia	No	0 (0.0)	12679	97.6	97.6	0 (0.0)	2917	97.1	97.1
	Yes		317	2.4	2.4		88	2.9	2.9
Preterm	No	0 (0.0)	12298	94.6	94.6	0 (0.0)	2837	94.4	94.4
	Yes		698	5.4	5.4		168	5.6	5.6
Small-for-gestational age	No	152 (1.2)	11567	90.1	90.1	4 (0.13)	2701	90.0	90.0
	Yes		1277	9.9	9.9		300	10.0	10.0
Maternal BMI		2179 (16.8)	10817	23.0 (3.9)	23.0 (3.8)	468 (15.6)	2537	24.9 (4.7)	24.9 (4.7)
	Underweight		549	5.1	5.9		54	2.1	2.7
	Normal		8014	74.1	72.4		1505	59.3	57.6
	Overweight		1642	15.2	16.4		646	25.5	26.6
	Obese		612	5.7	5.4		332	13.1	13.1
Maternal height		1536 (11.8)	11460	164.0 (6.7)	163.9 (6.7)	15 (0.5)	2990	163.2 (6.4)	163.2 (6.4)
Maternal age		0 (0.0)	12996	27.9 (5.0)	27.9 (5.0)	0 (0.0)	3005	30.6 (3.8)	30.6 (3.8)
	Up to 34 years		11722	90.2	90.2		2590	86.2	86.2
	35+ years		1274	9.8	9.8		415	13.8	13.8
Parity	Nulliparous	924 (7.1)	5509	45.6	45.9	2 (0.1)	1522	50.7	50.7
	1		4171	34.6	34.4		990	33.0	33.0
	2+		2392	19.8	19.7		491	16.4	16.3

Maternal smoking in pregnancy	No	795 (6.1)	9101	74.6	74.1	140 (4.7)	2394	83.6	83.4
	Yes		3100	25.4	25.9		471	16.4	16.6
Essential hypertension	No	1555 (12.0)	11011	96.2	96.3	293 (9.8)	2644	97.5	97.4
	Yes		430	3.8	3.7		68	2.5	2.6
Previous gestational hypertension	No	1073 (8.3)	10900	91.4	91.0	1626 (54.1)	1215	88.1	62.4
	Yes		1023	8.6	9.0		164	11.9	37.6
Diabetes	No	1380 (10.6)	11573	99.6	99.6	294 (9.8)	2699	99.6	99.4
	Yes		43	0.4	0.4		12	0.4	0.6
Previous gestational diabetes	No	991 (7.6)	11947	99.5	99.5	1627 (54.1)	1361	98.8	91.7
	Yes		58	0.5	0.5		17	1.2	8.3
Maternal ethnicity	White	1426 (11.0)	11265	97.4	97.2	0 (0.0)	2868	95.4	95.4
	Non-white		305	2.6	2.8		137	4.6	4.6

Table E Mean arterial pressure in the observed and imputed datasets in ALSPAC and the SWS

Blood pressure (mm Hg)	ALSPAC					SWS				
	N not yet delivered	N (%) missing	N with data	Observed Mean (SD)	Imputed Mean (SD)	N not yet delivered	N (%) missing	N with data	Observed Mean (SD)	Imputed Mean (SD)
Initial MAP	12996	919 (7.1)	12077	81.3 (8.8)	81.3 (8.8)	3005	114 (3.8)	2891	81.9 (8.7)	81.9 (8.7)
MAP at 20 weeks	12996	1759 (13.5)	11237	80.3 (8.5)	80.3 (8.5)	3005	317 (10.5)	2688	80.3 (8.4)	80.2 (8.4)
MAP at 25 weeks	12989	1432 (11.0)	11557	80.9 (8.5)	80.9 (8.5)	3005	213 (7.1)	2792	81.2 (8.4)	81.2 (8.4)
MAP at 28 weeks	12965	884 (6.8)	12081	81.4 (8.6)	81.4 (8.6)	2999	81 (2.7)	2918	81.8 (8.5)	81.7 (8.5)
MAP at 31 weeks	12907	657 (5.1)	12250	82.2 (8.7)	82.1 (8.7)	2988	69 (2.3)	2919	82.7 (8.7)	82.6 (8.7)
MAP at 34 weeks	12789	366 (2.9)	12423	83.0 (9.2)	83.0 (9.2)	2965	32 (1.1)	2933	83.5 (9.2)	83.5 (9.2)
MAP at 36 weeks	12565	329 (2.6)	12236	84.4 (9.7)	84.3 (9.7)	2903	19 (0.7)	2884	84.8 (9.3)	84.8 (9.3)

Table F Unadjusted associations of each of the potential predictor variables with preeclampsia, preterm birth and small-for-gestational age in ALSPAC (N=12996) and SWS (N=3005)

		Preeclampsia		Preterm birth		Small-for-gestational age	
		ALSPAC	SWS	ALSPAC	SWS	ALSPAC	SWS
		Odds ratio (95% CI)	Odds ratio (95% CI)	Odds ratio (95% CI)	Odds ratio (95% CI)	Odds ratio (95% CI)	Odds ratio (95% CI)
Initial MAP (mmHg)		1.07 (1.06, 1.09)	1.05 (1.02, 1.07)	1.00 (0.99, 1.01)	1.02 (1.00, 1.04)	0.99 (0.99, 1.00)	0.99 (0.98, 1.00)
MAP at 20 weeks (mmHg)		1.09 (1.08, 1.11)	1.07 (1.04, 1.10)	1.00 (1.00, 1.01)	1.02 (1.00, 1.04)	0.99 (0.99, 1.00)	0.99 (0.98, 1.01)
MAP at 25 weeks (mmHg)		1.11 (1.10, 1.12)	1.08 (1.05, 1.10)	1.02 (1.01, 1.03)	1.03 (1.01, 1.05)	0.99 (0.98, 1.00)	0.99 (0.97, 1.00)
MAP at 28 weeks (mmHg)		1.11 (1.10, 1.13)	1.12 (1.09, 1.15)	1.03 (1.02, 1.04)	1.04 (1.02, 1.06)	1.00 (0.99, 1.00)	1.01 (0.99, 1.02)
MAP at 31 weeks (mmHg)		1.12 (1.11, 1.13)	1.13 (1.11, 1.16)	1.05 (1.04, 1.06)	1.05 (1.04, 1.07)	1.01 (1.00, 1.02)	1.01 (1.00, 1.03)
MAP at 34 weeks (mmHg)		1.13 (1.12, 1.14)	1.14 (1.11, 1.16)	1.07 (1.07, 1.08)	1.07 (1.06, 1.09)	1.02 (1.01, 1.02)	1.01 (1.00, 1.03)
MAP at 36 weeks (mmHg)		1.14 (1.13, 1.15)	1.15 (1.13, 1.18)	1.08 (1.07, 1.09)	1.07 (1.05, 1.08)	1.01 (1.01, 1.02)	1.01 (1.00, 1.02)
Maternal BMI (kg/m^2)		1.10 (1.07, 1.12)	1.05 (1.01, 1.10)	0.99 (0.97, 1.01)	0.99 (0.96, 1.03)	0.95 (0.93, 0.96)	0.94 (0.91, 0.97)
Maternal height (cm)		0.98 (0.96, 1.00)	1.01 (0.98, 1.04)	0.97 (0.96, 0.98)	0.98 (0.95, 1.00)	0.95 (0.94, 0.96)	0.93 (0.92, 0.95)
Maternal age over 35 years		1.42 (1.02, 1.97)	0.70 (0.35, 1.42)	0.98 (0.75, 1.26)	0.84 (0.52, 1.35)	0.90 (0.74, 1.10)	1.08 (0.77, 1.51)
Maternal parity	0 (ref)	1	1	1	1	1	1
	1	0.33 (0.24, 0.45)	0.16 (0.08, 0.33)	0.65 (0.54, 0.79)	0.77 (0.54, 1.10)	0.48 (0.42, 0.55)	0.42 (0.31, 0.56)

	2 or more	0.39 (0.27, 0.56)	0.20 (0.08, 0.49)	0.88 (0.71, 1.08)	0.73 (0.46, 1.17)	0.50 (0.42, 0.60)	0.51 (0.35, 0.74)
Maternal smoking in pregnancy		0.53 (0.38, 0.73)	0.78 (0.41, 1.48)	1.11 (0.94, 1.33)	1.64 (1.11, 2.41)	2.32 (2.04, 2.64)	2.63 (2.00, 3.46)
Pre-existing hypertension		3.57 (2.44, 5.24)	5.39 (2.58, 11.25)	1.52 (1.06, 2.18)	2.82 (1.40, 5.66)	1.34 (1.00, 1.80)	2.02 (1.05, 3.90)
Previous gestational hypertension		1.86 (1.35, 2.58)	8.97 (2.86, 28.14)	1.20 (0.91, 1.58)	1.75 (1.13, 2.71)	0.74 (0.58, 0.94)	1.85 (0.99, 3.47)
Pre-existing diabetes		12.12 (5.95, 24.69)	6.01 (1.34, 27.07)	7.72 (3.71, 16.08)	5.88 (1.78, 19.45)	1.16 (0.45, 2.97)	1.16 (0.17, 7.91)
Previous gestational diabetes		2.24 (0.67, 7.48)	3.37 (0.39, 28.91)	2.99 (1.40, 6.37)	2.04 (0.53, 7.95)	0.17 (0.02, 1.32)	1.00 (0.13, 7.75)
Maternal non-white ethnicity		1.55 (0.85, 2.85)	0.48 (0.12, 1.97)	2.32 (1.54, 3.49)	1.67 (0.90, 3.08)	2.60 (1.96, 3.43)	2.22 (1.42, 3.46)

Table G AUROCs for prediction models for preeclampsia from Models 1-4 at different gestational ages in ALSPAC and the SWS*

Gestational age of prediction (X)	Development cohort – ALSPAC<br>AUROC (95% CI)				Validation cohort – SWS<br>AUROC (95% CI)			
	**Model 1**	**Model 2**	**Model 3**	**Model 4**	**Model 1**	**Model 2**	**Model 3**	**Model 4**
20 weeks	0.77 (0.75, 0.80)	0.79 (0.77, 0.82)	0.79 (0.77, 0.82)	0.79 (0.77, 0.82)	0.79 (0.73, 0.85)	0.80 (0.74, 0.85)	0.80 (0.74, 0.85)	0.80 (0.74, 0.85)
		P vs M1 = 0.002	P vs M1 = 0.001	P vs M2 = 0.20		P vs M1 = 0.47	P vs M1 = 0.49	P vs M2 = 0.63
25 weeks	0.77 (0.75, 0.80)	0.80 (0.77, 0.82)	0.80 (0.78, 0.83)	0.80 (0.78, 0.83)	0.79 (0.73, 0.85)	0.80 (0.75, 0.86)	0.80 (0.75, 0.86)	0.80 (0.75, 0.86)
		P vs M1 < 0.001	P vs M1 < 0.001	P vs M2 = 0.16		P vs M1 = 0.39	P vs M1 = 0.38	P vs M2 = 0.91
28 weeks	0.77 (0.75, 0.80)	0.81 (0.79, 0.84)	0.81 (0.79, 0.84)	0.81 (0.79, 0.84)	0.79 (0.73, 0.85)	0.84 (0.79, 0.88)	0.83 (0.78, 0.88)	0.83 (0.78, 0.88)
		P vs M1 < 0.001	P vs M1 < 0.001	P vs M2 = 0.42		P vs M1 = 0.003	P vs M1 = 0.004	P vs M2 = 0.65
31 weeks	0.77 (0.74, 0.79)	0.82 (0.80, 0.85)	0.83 (0.80, 0.85)	0.83 (0.80, 0.85)	0.79 (0.73, 0.85)	0.84 (0.79, 0.89)	0.84 (0.79, 0.89)	0.84 (0.79, 0.89)
		P vs M1 < 0.001	P vs M1 < 0.001	P vs M2 = 0.37		P vs M1 = 0.008	P vs M1 = 0.009	P vs M2 = 0.83
34 weeks	0.77 (0.74, 0.80)	0.84 (0.82, 0.87)	0.84 (0.82, 0.87)	0.84 (0.82, 0.87)	0.81 (0.75, 0.87)	0.86 (0.81, 0.90)	0.86 (0.81, 0.90)	0.86 (0.81, 0.90)
		P vs M1 < 0.001	P vs M1 < 0.001	P vs M2 = 0.29		P vs M1 = 0.01	P vs M1 = 0.01	P vs M2 = 0.53
36 weeks	0.77 (0.74, 0.80)	0.88 (0.86, 0.90)	0.88 (0.86, 0.90)	0.88 (0.86, 0.90)	0.80 (0.74, 0.87)	0.88 (0.84, 0.93)	0.88 (0.84, 0.92)	0.88 (0.84, 0.92)
		P vs M1 < 0.001	P vs M1 < 0.001	P vs M2 = 0.15		P vs M1 < 0.001	P vs M1 < 0.001	P vs M2 = 0.57

* Model 1 includes maternal covariates and MAP at the first visit; Model 2 is as Model 1 plus the observed value of MAP at X weeks; Model 3 is as Model 1 plus the deviation of the observed value of MAP from the normogram prediction at X weeks; Model 4 is as Model 1 plus both the observed value of MAP at X weeks and the deviation of the observed value of MAP from the normogram prediction at X weeks

Table H Calibration of the risk score for preeclampsia from Model 2 at 28 weeks gestation in the Southampton Women's Survey (N=2999)*

Tenth of prediction score	Original scale			Recalibrated		
	Prediction score values		Proportion who had preeclampsia (95% CI)	Prediction score values		Proportion who had preeclampsia (95% CI)
	Mean	Range		Mean	Range	
1	0.002	0.000 to 0.003	0.000	0.002	(0.000, 0.002)	0.000
2	0.004	0.003 to 0.005	0.001	0.003	(0.002, 0.003)	0.001
3	0.006	0.005 to 0.008	0.000	0.004	(0.003, 0.005)	0.000
4	0.010	0.008 to 0.013	0.006	0.006	(0.005, 0.008)	0.006
5	0.016	0.013 to 0.021	0.011	0.010	(0.008, 0.012)	0.011
6	0.027	0.021 to 0.033	0.018	0.015	(0.012, 0.018)	0.018
7	0.042	0.033 to 0.052	0.025	0.023	(0.018, 0.027)	0.025
8	0.067	0.052 to 0.086	0.040	0.035	(0.027, 0.043)	0.040
9	0.117	0.086 to 0.158	0.057	0.057	(0.043, 0.076)	0.057
10	0.285	0.158 to 0.788	0.132	0.144	(0.077, 0.540)	0.132

* Model 2 includes maternal covariates, MAP at the first visit and MAP at 28 weeks

Table I Prediction of preeclampsia in ALSPAC and SWS using Model 2 at 28 weeks gestation for fixed specificities*

Specificity	Risk score threshold	Sensitivity (95% CI)	Positive predictive value (95% CI)	Negative predictive value (95% CI)	Positive likelihood ratio (95% CI)	Negative likelihood ratio (95% CI)	N (%) testing positive	N (%) true positive	N (%) false positive	N (%) true negative	N (%) false negative
**ALSPAC**											
0.75	0.027	0.71 (0.66, 0.76)	0.07 (0.06, 0.07)	0.99 (0.99, 0.99)	2.84 (2.62, 3.08)	0.39 (0.32, 0.47)	3384 (26.1)	222 (1.7)	3162 (24.4)	9490 (73.2)	91 (0.7)
0.90	0.053	0.48 (0.42, 0.54)	0.11 (0.09, 0.12)	0.99 (0.98, 0.99)	4.78 (4.19, 5.46)	0.58 (0.52, 0.65)	1413 (10.9)	150 (1.2)	1264 (9.7)	11388 (87.8)	164 (1.3)
0.95	0.078	0.35 (0.29, 0.40)	0.15 (0.12, 0.17)	0.98 (0.98, 0.99)	6.93 (5.8, 8.27)	0.69 (0.63, 0.75)	740 (5.7)	108 (0.8)	632 (4.9)	12020 (92.7)	205 (1.6)
**SWS (without recalibration, using ALSPAC risk score thresholds)**											
0.58 (0.49, 0.67)	0.027	0.90 (0.80, 1.00)	0.06 (0.04, 0.08)	1.00 (0.99, 1.00)	2.16 (1.79, 2.61)	0.16 (0.06, 0.40)	1298 (43.3)	78 (2.6)	1219 (40.6)	1693 (56.5)	9 (0.3)
0.73 (0.62, 0.84)	0.053	0.79 (0.64, 0.95)	0.08 (0.05, 0.11)	0.99 (0.99, 1.00)	2.93 (2.18, 3.94)	0.27 (0.12, 0.60)	866 (28.9)	69 (2.3)	797 (26.6)	2115 (70.5)	18 (0.6)
0.80 (0.69, 0.91)	0.078	0.66 (0.47, 0.85)	0.09 (0.06, 0.13)	0.99 (0.98, 0.99)	3.36 (2.36, 4.78)	0.41 (0.24, 0.72)	641 (21.4)	57 (1.9)	583 (19.4)	2329 (77.7)	30 (1.0)
**SWS (after recalibration)**											
0.75	0.034	0.76 (0.62, 0.90)	0.08 (0.06, 0.11)	0.99 (0.98, 1.00)	3.03 (2.49, 3.69)	0.31 (0.16, 0.60)	794 (26.5)	66 (2.2)	728 (24.3)	2184 (72.8)	21 (0.7)
0.90	0.075	0.48 (0.35, 0.62)	0.13 (0.08, 0.17)	0.98 (0.98, 0.99)	4.83 (3.54, 6.58)	0.57 (0.43, 0.75)	333 (11.1)	42 (1.4)	291 (9.7)	2621 (87.4)	45 (1.5)
0.95	0.114	0.32 (0.20, 0.43)	0.16 (0.10, 0.22)	0.98 (0.97, 0.98)	6.28 (4.22, 9.36)	0.72 (0.61, 0.85)	173 (5.8)	28 (0.9)	146 (4.9)	2766 (92.2)	60 (2.0)

* Model 2 includes maternal covariates, MAP at the first visit and the observed value of MAP at 28 weeks

Table J AUROCs for prediction models for preterm birth at different gestational ages in ALSPAC and the SWS*

Gestational age of prediction (X)	Development cohort – ALSPAC AUROC (95% CI)				Validation cohort – SWS AUROC (95% CI)			
	Model 1	Model 2	Model 3	Model 4	Model 1	Model 2	Model 3	Model 4
20 weeks	0.61 (0.58, 0.63)	0.61 (0.58, 0.63)	0.61 (0.58, 0.63)	0.61 (0.58, 0.63)	0.60 (0.55, 0.66)	0.61 (0.55, 0.66)	0.61 (0.55, 0.66)	0.62 (0.56, 0.67)
		P vs M1 = 0.99	P vs M1 = 0.97	P vs M2 = 0.55		P vs M1 = 0.42	P vs M1 = 0.28	P vs M2 = 0.006
25 weeks	0.60 (0.58, 0.63)	0.61 (0.59, 0.63)	0.61 (0.59, 0.63)	0.61 (0.59, 0.64)	0.60 (0.55, 0.66)	0.61 (0.56, 0.67)	0.61 (0.56, 0.67)	0.62 (0.57, 0.68)
		P vs M1 = 0.36	P vs M1 = 0.34	P vs M2 = 0.44		P vs M1 = 0.26	P vs M1 = 0.21	P vs M2 = 0.03
28 weeks	0.60 (0.58, 0.63)	0.61 (0.59, 0.64)	0.61 (0.59, 0.64)	0.62 (0.59, 0.64)	0.61 (0.55, 0.66)	0.61 (0.56, 0.67)	0.61 (0.56, 0.67)	0.62 (0.57, 0.68)
		P vs M1 = 0.13	P vs M1 = 0.12	P vs M2 = 0.35		P vs M1 = 0.65	P vs M1 = 0.54	P vs M2 = 0.02
31 weeks	0.60 (0.57, 0.62)	0.63 (0.60, 0.65)	0.63 (0.60, 0.65)	0.63 (0.60, 0.65)	0.59 (0.54, 0.65)	0.60 (0.55, 0.66)	0.60 (0.55, 0.66)	0.61 (0.56, 0.66)
		P vs M1 = 0.005	P vs M1 = 0.004	P vs M2 = 0.44		P vs M1 = 0.72	P vs M1 = 0.62	P vs M2 = 0.03
34 weeks	0.60 (0.57, 0.62)	0.66 (0.63, 0.69)	0.66 (0.63, 0.69)	0.66 (0.63, 0.69)	0.58 (0.52, 0.64)	0.65 (0.59, 0.71)	0.65 (0.59, 0.71)	0.65 (0.59, 0.71)
		P vs M1 < 0.001	P vs M1 < 0.001	P vs M2 = 0.71		P vs M1 = 0.01	P vs M1 = 0.009	P vs M2 = 0.07
36 weeks	0.58 (0.54, 0.62)	0.69 (0.65, 0.73)	0.69 (0.65, 0.72)	0.69 (0.65, 0.73)	0.53 (0.45, 0.61)	0.59 (0.51, 0.67)	0.59 (0.51, 0.67)	0.58 (0.51, 0.66)
		P vs M1 < 0.001	P vs M1 < 0.001	P vs M2 = 0.57		P vs M1 = 0.17	P vs M1 = 0.15	P vs M2 = 0.12

* Model 1 includes maternal covariates and MAP at the first visit; Model 2 is as Model 1 plus the observed value of MAP at X weeks; Model 3 is as Model 1 plus the deviation of the observed value of MAP from the normogram prediction at X weeks; Model 4 is as Model 1 plus both the observed value of MAP at X weeks and the deviation of the observed value of MAP from the normogram prediction at X weeks
M1: Model 1; M2: Model 2

Table K AUROCs for prediction models for preterm birth at different gestational ages in ALSPAC and the SWS excluding women who developed preeclampsia*

Gestational age of prediction (X)	Development cohort – ALSPAC AUROC (95% CI)				Validation cohort – SWS AUROC (95% CI)			
	Model 1	Model 2	Model 3	Model 4	Model 1	Model 2	Model 3	Model 4
20 weeks	0.60 (0.57, 0.62)	0.60 (0.57, 0.62)	0.60 (0.57, 0.62)	0.60 (0.57, 0.62)	0.57 (0.52, 0.63)	0.57 (0.51, 0.63)	0.57 (0.52, 0.63)	0.58 (0.53, 0.64)
		P vs M1 = 0.04	P vs M1 < 0.001	P vs M2 = 0.59		P vs M1 = 0.36	P vs M1 = 0.19	P vs M2 = 0.01
25 weeks	0.59 (0.57, 0.62)	0.60 (0.57, 0.62)	0.60 (0.57, 0.62)	0.60 (0.57, 0.62)	0.57 (0.52, 0.63)	0.57 (0.52, 0.63)	0.57 (0.52, 0.63)	0.59 (0.53, 0.65)
		P vs M1 = 0.72	P vs M1 = 0.69	P vs M2 = 0.68		P vs M1 = 0.75	P vs M1 = 0.74	P vs M2 = 0.02
28 weeks	0.59 (0.57, 0.62)	0.60 (0.57, 0.62)	0.60 (0.57, 0.62)	0.60 (0.58, 0.63)	0.58 (0.52, 0.63)	0.57 (0.52, 0.63)	0.57 (0.52, 0.63)	0.59 (0.53, 0.65)
		P vs M1 = 0.24	P vs M1 = 0.22	P vs M2 = 0.50		P vs M1 = 0.61	P vs M1 = 0.75	P vs M2 = 0.01
31 weeks	0.59 (0.57, 0.62)	0.61 (0.59, 0.64)	0.62 (0.59, 0.64)	0.62 (0.59, 0.64)	0.57 (0.51, 0.62)	0.57 (0.51, 0.62)	0.57 (0.51, 0.62)	0.58 (0.52, 0.64)
		P vs M1 = 0.03	P vs M1 = 0.03	P vs M2 = 0.47		P vs M1 = 0.92	P vs M1 = 0.99	P vs M2 = 0.02
34 weeks	0.59 (0.56, 0.62)	0.64 (0.61, 0.67)	0.64 (0.61, 0.67)	0.64 (0.61, 0.67)	0.56 (0.49, 0.62)	0.62 (0.55, 0.68)	0.62 (0.55, 0.68)	0.62 (0.56, 0.69)
		P vs M1 < 0.001	P vs M1 < 0.001	P vs M2 = 0.65		P vs M1 = 0.02	P vs M1 = 0.02	P vs M2 = 0.11
36 weeks	0.57 (0.53, 0.61)	0.67 (0.63, 0.70)	0.66 (0.63, 0.70)	0.67 (0.63, 0.70)	0.53 (0.45, 0.61)	0.59 (0.51, 0.67)	0.59 (0.51, 0.67)	0.58 (0.50, 0.66)
		P vs M1 < 0.001	P vs M1 < 0.001	P vs M2 = 0.52		P vs M1 = 0.17	P vs M1 = 0.15	P vs M2 = 0.10

* Model 1 includes maternal covariates and MAP at the first visit; Model 2 is as Model 1 plus the observed value of MAP at X weeks; Model 3 is as Model 1 plus the deviation of the observed value of MAP from the normogram prediction at X weeks; Model 4 is as Model 1 plus both the observed value of MAP at X weeks and the deviation of the observed value of MAP from the normogram prediction at X weeks
M1: Model 1; M2: Model 2

Table L AUROCs for prediction models for small for gestational age at different gestational ages in ALSPAC and the SWS*

Gestational age of prediction (X)	Development cohort – ALSPAC AUROC (95% CI)				Validation cohort – SWS AUROC (95% CI)			
	**Model 1**	**Model 2**	**Model 3**	**Model 4**	**Model 1**	**Model 2**	**Model 3**	**Model 4**
20 weeks	0.70 (0.68, 0.71)	0.70 (0.68, 0.71)	0.70 (0.68, 0.71)	0.70 (0.68, 0.71)	0.70 (0.61, 0.79)	0.70 (0.61, 0.79)	0.70 (0.61, 0.79)	0.70 (0.61, 0.79)
		P vs M1 < 0.001	P vs M1 < 0.001	P vs M2 = 0.38		P vs M1 = 0.59	P vs M1 = 0.76	P vs M2 = 0.30
25 weeks	0.70 (0.68, 0.71)	0.70 (0.68, 0.71)	0.70 (0.68, 0.71)	0.70 (0.68, 0.71)	0.70 (0.61, 0.79)	0.70 (0.62, 0.79)	0.70 (0.61, 0.79)	0.70 (0.62, 0.79)
		P vs M1 = 0.15	P vs M1 = 0.13	P vs M2 = 0.51		P vs M1 = 0.55	P vs M1 = 0.55	P vs M2 = 0.78
28 weeks	0.70 (0.68, 0.71)	0.70 (0.68, 0.71)	0.70 (0.68, 0.71)	0.70 (0.68, 0.71)	0.71 (0.62, 0.80)	0.71 (0.62, 0.79)	0.71 (0.62, 0.79)	0.71 (0.62, 0.79)
		P vs M1 = 0.14	P vs M1 = 0.14	P vs M2 = 0.73		P vs M1 = 0.35	P vs M1 = 0.31	P vs M2 = 0.95
31 weeks	0.70 (0.68, 0.71)	0.70 (0.68, 0.71)	0.70 (0.68, 0.71)	0.70 (0.68, 0.71)	0.70 (0.61, 0.79)	0.70 (0.61, 0.79)	0.70 (0.61, 0.79)	0.70 (0.61, 0.79)
		P vs M1 = 0.14	P vs M1 = 0.14	P vs M2 = 0.84		P vs M1 = 0.87	P vs M1 = 0.85	P vs M2 = 0.52
34 weeks	0.70 (0.68, 0.71)	0.70 (0.68, 0.72)	0.70 (0.68, 0.72)	0.70 (0.68, 0.72)	0.70 (0.61, 0.79)	0.70 (0.61, 0.79)	0.70 (0.61, 0.79)	0.70 (0.61, 0.78)
		P vs M1 = 0.07	P vs M1 = 0.07	P vs M2 = 0.76		P vs M1 = 0.93	P vs M1 = 0.96	P vs M2 = 0.21
36 weeks	0.69 (0.68, 0.71)	0.70 (0.68, 0.71)	0.70 (0.68, 0.71)	0.70 (0.68, 0.71)	0.71 (0.62, 0.80)	0.71 (0.62, 0.80)	0.71 (0.62, 0.80)	0.71 (0.62, 0.80)
		P vs M1 = 0.09	P vs M1 = 0.09	P vs M2 = 0.77		P vs M1 = 0.92	P vs M1 = 0.87	P vs M2 = 0.11

* Model 1 includes maternal covariates and MAP at the first visit; Model 2 is as Model 1 plus the observed value of MAP at X weeks; Model 3 is as Model 1 plus the deviation of the observed value of MAP from the normogram prediction at X weeks; Model 4 is as Model 1 plus both the observed value of MAP at X weeks and the deviation of the observed value of MAP from the normogram prediction at X weeks

M1: Model 1; M2: Model 2

Table M Risk factors included in clinical risk prediction model for preeclampsia in the SCOPE study

**Maternal clinical risk factors model (North et al, 2011)[15]**
Age
MAP
BMI
Family history of preeclampsia
Family history of coronary heart disease
Woman's birth weight
Vaginal bleeding ≥ 5 days
One miscarriage ≤ 10 weeks, same partner
≥ 12 months to conceive
High fruit intake (≥ 3/day)
Alcohol consumption 1st trimester
Number of cigarettes smoked per day
