# Supplementary material for: Antenatal blood pressure for prediction of pre-eclampsia, preterm birth, and small for gestational age babies: development and validation in two general population cohorts
Source: BMJ. 2015 Nov 17;351:h5948. doi: 10.1136/bmj.h5948 (PMC4647185; doi:10.1136/bmj.h5948)
Supplement: Supplementary file 2 [file macc026935.ww2_default.pdf]

Appendix 2 Supplemental Methods [posted as supplied by author]

Prediction of blood pressure at different gestational ages from normograms

To prevent women who had many measurements of blood pressure during pregnancy from having too high an influence on the models, we randomly selected one blood pressure measurement per woman for any 2-week period where the woman had more than one measurement for inclusion in the models. This led to a median and interquartile range of 10 (9 to 11) blood pressure measurements per woman included in the models.

We included pre-pregnancy BMI in the multilevel models in four categories: underweight (<18.5 kg/m^2), normal weight (18.5-24.9 kg/m^2), overweight (25-29.9 kg/m^2) and obese (≥30 kg/m^2) as a covariate and also included an interaction between BMI category and each of the splines to allow the shape of the BP trajectory to differ by BMI category. Smoking (any smoking or never smoked) was also included as a categorical covariate and as an interaction with each of the splines. We fitted separate models for nulliparous and multiparous women.

The equation of the multilevel model for blood pressure change across pregnancy was:

$$
\begin{aligned}
y_{ij} = {} & \beta_0 + u_{0j} + (\beta_1 + u_{1j})spline1_{ij} + (\beta_2 + u_{2j})spline2_{ij} + (\beta_3 + u_{3j})spline3_{ij} + (\beta_4 + u_{4j})spline4_{ij} + \\
& \beta_5 underweight_j + \beta_6 overweight_j + \beta_7 obese_j + \\
& \beta_8 underweight_j \times spline1_{ij} + \beta_9 overweight_j \times spline1_{ij} + \beta_{10} obese_j \times spline1_{ij} + \\
& \beta_{11} underweight_j \times spline2_{ij} + \beta_{12} overweight_j \times spline2_{ij} + \beta_{13} obese_j \times spline2_{ij} + \\
& \beta_{14} underweight_j \times spline3_{ij} + \beta_{15} overweight_j \times spline3_{ij} + \beta_{16} obese_j \times spline3_{ij} + \\
& \beta_{17} underweight_j \times spline4_{ij} + \beta_{18} overweight_j \times spline4_{ij} + \beta_{19} obese_j \times spline4_{ij} + \\
& \beta_{20} smoking_j + \beta_{21} smoking_j \times spline1_{ij} + \beta_{22} smoking_j \times spline2_{ij} + \beta_{23} smoking_j \times spline3_{ij} + \\
& \beta_{24} smoking_j \times spline4_{ij} + \\
& e_{0ij} + e_{1ij}(GA_{ij} - 12)
\end{aligned}
$$

$$\begin{bmatrix} u_{0j} \\ u_{1j} \\ u_{2j} \\ u_{3j} \\ u_{4j} \end{bmatrix} \sim N(\mathbf{0}, \Omega_u), \begin{bmatrix} e_{0ij} \\ e_{1ij} \end{bmatrix} \sim N(\mathbf{0}, \Omega_e)$$

where, y_{ij} is the value of the ith MAP measurement on the jth individual, β_0-β_{24} describe the average trajectory of change, u_{0j}-u_{4j} describe how the jth individual's trajectory of MAP deviates from the average and GA_{ij} is the gestational age in weeks of the ith measurement on the jth individual. It is centred at 12 weeks in order to set the intercept, β_0, to represent blood pressure at 12 weeks. The e_{0ij} and e_{1ij} terms describe the deviation of the ith measurement of MAP on the jth individual from the individual's trajectory. These are residual error terms.

The model was fitted separately for nulliparous and multiparous women rather than including parity as additional covariate, to allow for greater flexibility in the shape and variability of the trajectories for nulliparous and multiparous women.

The splines are defined as: (1)

$$spline1_{ij} = GA_{ij} - 12$$

$$spline\,2_{ij} = \frac{(GA_{ij} - 11)_+ - (40-36)^{-1}\{(GA_{ij} - 36)_+^3(40-11) - (GA_{ij} - 40)_+^3(36-11)\}}{(40-11)^2}$$

$$spline\,3_{ij} = \frac{(GA_{ij} - 18)_+ - (40-36)^{-1}\{(GA_{ij} - 36)_+^3(40-18) - (GA_{ij} - 40)_+^3(36-18)\}}{(40-11)^2}$$

$$spline\,4_{ij} = \frac{(GA_{ij} - 30)_+ - (40-36)^{-1}\{(GA_{ij} - 36)_+^3(40-30) - (GA_{ij} - 40)_+^3(36-30)\}}{(40-11)^2}$$

where $(x)_+ = \begin{cases} 0 \text{ if } x \le 0 \\ x \text{ if } x > 0 \end{cases}$

To calculate predictions conditional on the initial blood pressure measurement we used the multilevel models as above, and applied the method described by Tilling et al(2) and Pan and Goldstein.(3)

The between-individual variance for individual j at the initial visit is:

$$V_{b1j} = \begin{bmatrix} 1 \\ spline1_{1j} \\ spline2_{1j} \\ spline3_{1j} \\ spline4_{1j} \end{bmatrix}^T \Omega_u \begin{bmatrix} 1 \\ spline1_{1j} \\ spline2_{1j} \\ spline3_{1j} \\ spline4_{1j} \end{bmatrix}$$

The within-individual variance for individual j at the initial visit is:

$$V_{w1} = \begin{bmatrix} 1 \\ GA_{1j} - 12 \end{bmatrix}^T \Omega_e \begin{bmatrix} 1 \\ GA_{1j} - 12 \end{bmatrix}.$$

The covariance between the deviations from the predicted curve at the initial visit (indexed 1) and g weeks for the jth individual is: $V_{b1,gj} = \begin{bmatrix} 1 \\ spline1_{1j} \\ spline2_{1j} \\ spline3_{1j} \\ spline4_{1j} \end{bmatrix}^T \Omega_u \begin{bmatrix} 1 \\ spline1_{gj} \\ spline2_{gj} \\ spline3_{gj} \\ spline4_{gj} \end{bmatrix}$

From these, the predicted blood pressure at gestational age g for individual j, conditional on the blood pressure value at the initial visit can be calculated as:

$$
\begin{aligned}
y_{gj} = \frac{\hat{V}_{b1,gj}}{\hat{V}_{b1j} + \hat{V}_{w1j}} (y_{1j} - \hat{\beta}_0 + \hat{\beta}_1 spline1_1 + \hat{\beta}_2 spline2_1 + \hat{\beta}_3 spline3_1 + \hat{\beta}_4 spline4_1 + \\
\hat{\beta}_5 underweight_j + \hat{\beta}_6 overweight_j + \hat{\beta}_7 obese_j + \\
\hat{\beta}_8 underweight_j \times spline1_{1j} + \hat{\beta}_9 overweight_j \times spline1_{1j} + \hat{\beta}_{10} obese_j \times spline1_{1j} + \\
\hat{\beta}_{11} underweight_j \times spline2_{1j} + \hat{\beta}_{12} overweight_j \times spline2_{1j} + \hat{\beta}_{13} obese_j \times spline2_{1j} + \\
\hat{\beta}_{14} underweight_j \times spline3_{1j} + \hat{\beta}_{15} overweight_j \times spline3_{1j} + \hat{\beta}_{16} obese_j \times spline3_{1j} + \\
\hat{\beta}_{17} underweight_j \times spline4_{1j} + \hat{\beta}_{18} overweight_j \times spline4_{1j} + \hat{\beta}_{19} obese_j \times spline4_{1j} + \\
\hat{\beta}_{20} smoking_j + \hat{\beta}_{21} smoking_j \times spline1_{1j} + \hat{\beta}_{22} smoking_j \times spline2_{1j} + \hat{\beta}_{23} smoking_j \times spline3_{1j} + \\
\hat{\beta}_{24} smoking_j \times spline4_{1j}) + \\
\hat{\beta}_0 + \hat{\beta}_1 spline1_{gj} + \hat{\beta}_2 spline2_{gj} + \hat{\beta}_3 spline3_{gj} + \hat{\beta}_4 spline4_{gj} + \\
\hat{\beta}_5 underweight_j + \hat{\beta}_6 overweight_j + \hat{\beta}_7 obese_j + \\
\hat{\beta}_8 underweight_j \times spline1_{gj} + \hat{\beta}_9 overweight_j \times spline1_{gj} + \hat{\beta}_{10} obese_j \times spline1_{gj} + \\
\hat{\beta}_{11} underweight_j \times spline2_{gj} + \hat{\beta}_{12} overweight_j \times spline2_{gj} + \hat{\beta}_{13} obese_j \times spline2_{gj} + \\
\hat{\beta}_{14} underweight_j \times spline3_{gj} + \hat{\beta}_{15} overweight_j \times spline3_{gj} + \hat{\beta}_{16} obese_j \times spline3_{gj} + \\
\hat{\beta}_{17} underweight_j \times spline4_{gj} + \hat{\beta}_{18} overweight_j \times spline4_{gj} + \hat{\beta}_{19} obese_j \times spline4_{gj} + \\
\hat{\beta}_{20} smoking_j + \hat{\beta}_{21} smoking_j \times spline1_{gj} + \hat{\beta}_{22} smoking_j \times spline2_{gj} + \hat{\beta}_{23} smoking_j \times spline3_{gj} + \\
\hat{\beta}_{24} smoking_j \times spline4_{gj}
\end{aligned}
$$

Where y_{1j} is the observed value of the outcome (MAP) at the initial visit. Thus, the deviation of the initial measurement from the average trajectory was combined with the multilevel model information to predict what trajectory would be seen for the rest of gestation.

Multiple imputation of missing values in ALSPAC and the SWS

The same variables were included in multiple imputation models in both ALSPAC and SWS. A separate imputation was done for blood pressure at each gestational age: 20, 25, 28, 31, 34 and 36 weeks, imputing only to the number of women who still had not delivered at each gestation. The variables included were either exposures/covariates to include in prediction models, outcomes or predictors of missingness. The variables included and information about how they were included in multiple imputation models are shown in the table below:

Variable	Type of variable	Model used to predict missing data in this variable	How variable was entered in models to predict missing data in other variables
MAP at initial visit	Continuous	Linear regression	Continuous
MAP at X weeks	Continuous	Linear regression	Continuous
Preeclampsia	Binary	Logistic regression	Binary
Offspring small for gestational age	Binary	Logistic regression	Binary
Customised small for gestational age	Binary	Logistic regression	Binary
Gestational age at delivery	Continuous	No missing data	Continuous
Pre-existing maternal hypertension	Binary	Logistic regression	Binary
Previous gestational hypertension	Binary	Logistic regression	Binary

Previous gestational diabetes	Binary	Logistic regression	Binary
Pre-existing diabetes	Binary	Logistic regression	Binary
Maternal age	Continuous	Linear regression	Continuous
Parity	Ordered categorical (3 categories)	Ordinal logistic regression	2 indicator variables
Maternal weight	Skewed continuous	Log-linear regression	Continuous
Maternal height	Continuous	Linear regression	Continuous
Maternal smoking	Binary	Logistic regression	Binary
Maternal education	Ordered categorical (4 categories)	Ordinal logistic regression	3 indicator variables
Household social class	Ordered categorical (5 categories)	Ordinal logistic regression	4 indicator variables
Maternal non-white ethnicity	Binary	Logistic regression	Binary
Offspring sex	Binary	Logistic regression	Binary

Prediction Models for Preeclampsia:

The logistic regression models used to predict preeclampsia in ALSPAC are shown below. The risk of preeclampsia is calculated as $\frac{exp(\boldsymbol{\beta X})}{1+\exp(\boldsymbol{\beta X})}$ where $\boldsymbol{\beta X}$ is as follows:

map_1 = MAP at the initial visit

matbmi = Maternal pre-pregnancy BMI

matheight = Maternal height

mat_over 35 = Maternal age over 35 years

par2 = Parity of 1

par3 = Parity of 2 or more

matsmok = Maternal smoking in the first trimester

esshyp = Essential hypertension

prev_gesthyp = Gestational hypertension in a previous pregnancy

prev_diab = Existing diabetes

prev_gestdiab = Gestational diabetes in a previous pregnancy

mat_nonwhite = Maternal non-white ethnicity

Model 1

-3.46 + 0.046*map_1 + 0.057*matbmi - 0.024*matheight + 0.384*mat_over35 - 1.590*par2 - 1.546*par3 - 0.479*matsmok + 0.793*esshyp + 1.346*prev_gesthyp + 2.301*prev_diab + 0.941*prev_gestdiab + 0.429*mat_nonwhite

Model 2

20 weeks gestation: -3.57 + 0.060*map_20wks + 0.023*map_1 + 0.036*matbmi - 0.031*matheight + 0.319*mat_over35 - 1.535*par2 - 1.499*par3 - 0.446*matsmok + 0.545*esshyp + 1.162*prev_gesthyp + 2.208*prev_diab + 0.896*prev_gestdiab + 0.520*mat_nonwhite

25 weeks gestation: -3.65 + 0.077*map_25wks + 0.019*map_1 + 0.030*matbmi - 0.031*matheight + 0.308*mat_over35 - 1.447*par2 - 1.389*par3 - 0.411*matsmok + 0.484*esshyp + 1.130*prev_gesthyp + 2.089*prev_diab + 0.870*prev_gestdiab + 0.555*mat_nonwhite

28 weeks gestation: -3.75 + 0.087*map_28wks + 0.017*map_1 + 0.030*matbmi - 0.032*matheight + 0.383*mat_over35 - 1.448*par2 - 1.414*par3 - 0.398*matsmok + 0.356*esshyp + 0.977*prev_gesthyp + 2.143*prev_diab + 0.948*prev_gestdiab + 0.559*mat_nonwhite

31 weeks gestation: -3.79 + 0.088*map_31wks + 0.014*map_1 + 0.038*matbmi - 0.028*matheight + 0.385*mat_over35 - 1.420*par2 - 1.265*par3 - 0.523*matsmok + 0.339*esshyp + 0.855*prev_gesthyp + 1.857*prev_diab + 0.773*prev_gestdiab + 0.538*mat_nonwhite

34 weeks gestation: -3.93 + 0.092*map_34wks + 0.019*map_1 + 0.030*matbmi - 0.029*matheight + 0.330*mat_over35 - 1.306*par2 - 1.196*par3 - 0.590*matsmok + 0.222*esshyp + 0.746*prev_gesthyp + 1.460*prev_diab + 0.533*prev_gestdiab + 0.560*mat_nonwhite

36 weeks gestation: -4.32 + 0.113*map_36wks + 0.016*map_1 + 0.023*matbmi - 0.029*matheight + 0.136*mat_over35 - 1.166*par2 - 1.075*par3 - 0.411*matsmok - 0.134*esshyp + 0.550*prev_gesthyp + 1.621*prev_diab + 0.854*prev_gestdiab + 0.684*mat_nonwhite

Model 3

20 weeks gestation: -3.53 + 0.061*map_dev_20 + 0.043*map_1 + 0.054*matbmi - 0.031*matheight + 0.321*mat_over35 - 1.589*par2 - 1.553*par3 - 0.491*matsmok + 0.541*esshyp + 1.164*prev_gesthyp + 2.196*prev_diab + 0.901*prev_gestdiab + 0.531*mat_nonwhite

25 weeks gestation: -3.64 + 0.077*map_dev_25 + 0.043*map_1 + 0.049*matbmi - 0.031*matheight + 0.309*mat_over35 - 1.515*par2 - 1.456*par3 - 0.452*matsmok + 0.479*esshyp + 1.136*prev_gesthyp + 2.080*prev_diab + 0.882*prev_gestdiab + 0.566*mat_nonwhite

28 weeks gestation: -3.69 + 0.087*map_dev_28 + 0.042*map_1 + 0.049*matbmi - 0.033*matheight + 0.384*mat_over35 - 1.532*par2 - 1.496*par3 - 0.441*matsmok + 0.351*esshyp + 0.981*prev_gesthyp + 2.139*prev_diab + 0.964*prev_gestdiab + 0.568*mat_nonwhite

31 weeks gestation: -3.76 + 0.088*map_dev_31 + 0.039*map_1 + 0.059*matbmi - 0.028*matheight + 0.385*mat_over35 - 1.517*par2 - 1.361*par3 - 0.570*matsmok + 0.338*esshyp + 0.861*prev_gesthyp + 1.852*prev_diab + 0.790*prev_gestdiab + 0.545*mat_nonwhite

34 weeks gestation: -3.90 + 0.093*map_dev_34 + 0.046*map_1 + 0.060*matbmi - 0.029*matheight + 0.333*mat_over35 - 1.437*par2 - 1.330*par3 - 0.649*matsmok + 0.214*esshyp + 0.744*prev_gesthyp + 1.441*prev_diab + 0.544*prev_gestdiab + 0.575*mat_nonwhite

36 weeks gestation: -4.22 + 0.113*map_dev_36 + 0.050*map_1 + 0.073*matbmi - 0.029*matheight + 0.133*mat_over35 - 1.370*par2 - 1.294*par3 - 0.502*matsmok - 0.127*esshyp + 0.546*prev_gesthyp + 1.609*prev_diab + 0.831*prev_gestdiab + 0.702*mat_nonwhite

Prediction Models for Preterm Birth:

The logistic regression models used to predict preterm birth in ALSPAC are shown below. The risk of preterm birth is calculated as $\frac{exp(\boldsymbol{\beta X})}{1+\exp(\boldsymbol{\beta X})}$ where $\boldsymbol{\beta X}$ is as follows:

map_1 = MAP at the initial visit

norm_wt = normal weight

over_wt = overweight

obese = obese

par2 = Parity of 1

par3 = Parity of 2 or more

esshyp = Essential hypertension

matheight = Maternal height

prev_gesthyp = Gestational hypertension in a previous pregnancy

prev_diab = Existing diabetes

prev_gestdiab = Gestational diabetes in a previous pregnancy

mat_nonwhite = Maternal non-white ethnicity

Model 1

-2.39 - 0.003*map_1 - 0.439*norm_wt - 0.468*over_wt - 0.562*obese - 0.027*matheight - 0.501*par2 - 0.229*par3 + 0.497*esshyp + 0.403*prev_gesthyp + 2.026*prev_diab + 1.167*prev_gestdiab + 0.746*mat_nonwhite

Model 2

20 weeks gestation: -2.38 + 0.005*map_20wks - 0.005*map_1 - 0.447*norm_wt - 0.487*over_wt - 0.592*obese - 0.028*matheight - 0.496*par2 - 0.223*par3 + 0.474*esshyp + 0.387*prev_gesthyp + 2.016*prev_diab + 1.164*prev_gestdiab + 0.753*mat_nonwhite

25 weeks gestation: -2.37 + 0.023*map_25wks - 0.011*map_1 - 0.477*norm_wt - 0.549*over_wt - 0.679*obese - 0.029*matheight - 0.465*par2 - 0.191*par3 + 0.406*esshyp + 0.343*prev_gesthyp + 1.889*prev_diab + 1.148*prev_gestdiab + 0.776*mat_nonwhite

28 weeks gestation: -2.46 + 0.030*map_28wks - 0.014*map_1 - 0.451*norm_wt - 0.548*over_wt - 0.709*obese - 0.030*matheight - 0.447*par2 - 0.150*par3 + 0.363*esshyp + 0.311*prev_gesthyp + 1.908*prev_diab + 1.045*prev_gestdiab + 0.735*mat_nonwhite

31 weeks gestation: -2.59 + 0.049*map_31wks - 0.023*map_1 - 0.450*norm_wt - 0.542*over_wt - 0.748*obese - 0.031*matheight - 0.379*par2 - 0.082*par3 + 0.247*esshyp + 0.145*prev_gesthyp + 1.702*prev_diab + 1.057*prev_gestdiab + 0.711*mat_nonwhite

34 weeks gestation: -2.95 + 0.068*map_34wks - 0.024*map_1 - 0.422*norm_wt - 0.619*over_wt - 0.824*obese - 0.037*matheight - 0.222*par2 + 0.029*par3 + 0.014*esshyp + 0.038*prev_gesthyp + 1.195*prev_diab + 1.035*prev_gestdiab + 0.743*mat_nonwhite

36 weeks gestation: -3.86 + 0.076*map_36wks - 0.025*map_1 - 0.385*norm_wt - 0.567*over_wt - 0.748*obese - 0.046*matheight + 0.146*par2 + 0.230*par3 - 0.088*esshyp - 0.023*prev_gesthyp + 1.173*prev_diab + 0.723*prev_gestdiab + 0.873*mat_nonwhite

Model 3

20 weeks gestation: -2.39 + 0.006*map_dev_20 - 0.003*map_1 - 0.441*norm_wt - 0.468*over_wt - 0.563*obese - 0.028*matheight - 0.501*par2 - 0.228*par3 + 0.471*esshyp + 0.385*prev_gesthyp + 2.013*prev_diab + 1.164*prev_gestdiab + 0.754*mat_nonwhite

25 weeks gestation: -2.42 + 0.024*map_dev_25 - 0.004*map_1 - 0.439*norm_wt - 0.465*over_wt - 0.559*obese - 0.029*matheight - 0.488*par2 - 0.214*par3 + 0.402*esshyp + 0.341*prev_gesthyp + 1.882*prev_diab + 1.148*prev_gestdiab + 0.779*mat_nonwhite

28 weeks gestation: -2.50 + 0.031*map_dev_28 - 0.004*map_1 - 0.396*norm_wt - 0.437*over_wt - 0.552*obese - 0.030*matheight - 0.479*par2 - 0.182*par3 + 0.358*esshyp + 0.309*prev_gesthyp + 1.901*prev_diab + 1.046*prev_gestdiab + 0.738*mat_nonwhite

31 weeks gestation: -2.68 + 0.049*map_dev_31 - 0.008*map_1 - 0.369*norm_wt - 0.374*over_wt - 0.490*obese - 0.031*matheight - 0.437*par2 - 0.141*par3 + 0.244*esshyp + 0.144*prev_gesthyp + 1.695*prev_diab + 1.057*prev_gestdiab + 0.714*mat_nonwhite

34 weeks gestation: -3.06 + 0.068*map_dev_34 - 0.003*map_1 - 0.334*norm_wt - 0.397*over_wt - 0.395*obese - 0.037*matheight - 0.319*par2 - 0.071*par3 + 0.011*esshyp + 0.036*prev_gesthyp + 1.185*prev_diab + 1.031*prev_gestdiab + 0.748*mat_nonwhite

36 weeks gestation: -3.95 + 0.075*map_dev_36 - 0.003*map_1 - 0.292*norm_wt - 0.322*over_wt - 0.135*obese - 0.046*matheight + 0.014*par2 + 0.093*par3 - 0.089*esshyp - 0.023*prev_gesthyp + 1.165*prev_diab + 0.718*prev_gestdiab + 0.873*mat_nonwhite

Prediction Models for Small-For-Gestational Age:

The logistic regression models used to predict small-for-gestational age in ALSPAC are shown below. The risk of small-for-gestational age is calculated as $\frac{exp(\boldsymbol{\beta X})}{1+\exp(\boldsymbol{\beta X})}$ where $\boldsymbol{\beta X}$ is as follows:

map_1 = MAP at the initial visit

norm_wt = normal weight

over_wt= overweight

obese = obese

mat_over 35 = Maternal age over 35 years

par2 = Parity of 1

par3 = Parity of 2 or more

matsmok = Maternal smoking in the first trimester

esshyp = Essential hypertension

matheight = Maternal height

prev_gesthyp = Gestational hypertension in a previous pregnancy

prev_gestdiab = Gestational diabetes in a previous pregnancy

mat_nonwhite = Maternal non-white ethnicity

Model 1

-1.64 + 0.003*map_1 - 0.625*norm_wt - 0.791*over_wt - 1.072*obese - 0.058*matheight + 0.182*mat_over35 - 0.756*par2 - 0.824*par3 + 0.850*matsmok + 0.447*esshyp + 0.209*prev_gesthyp - 1.445*prev_gestdiab + 0.856*mat_nonwhite

Model 2

20 weeks gestation: -1.64 + 0.000*map_20wks + 0.003*map_1 - 0.626*norm_wt - 0.792* over_wt - 1.073*obese - 0.058*matheight + 0.182*mat_over35 - 0.756*par2 - 0.824*par3 + 0.851*matsmok + 0.446*esshyp + 0.208*prev_gesthyp - 1.445*prev_gestdiab + 0.857*mat_nonwhite

25 weeks gestation: -1.64 - 0.004*map_25wks + 0.005*map_1 - 0.620*norm_wt - 0.777* over_wt - 1.051*obese - 0.058*matheight + 0.186*mat_over35 - 0.762*par2 - 0.831*par3 + 0.847*matsmok + 0.463*esshyp + 0.219*prev_gesthyp -1.438*prev_gestdiab + 0.852*mat_nonwhite

28 weeks gestation: -1.63 + 0.003*map_28wks + 0.002*map_1 - 0.635*norm_wt - 0.804*over_wt - 1.093*obese - 0.058*matheight + 0.183*mat_over35 - 0.752*par2 - 0.821*par3 + 0.851*matsmok + 0.428*esshyp + 0.195*prev_gesthyp - 1.411*prev_gestdiab + 0.877*mat_nonwhite

31 weeks gestation: -1.63 + 0.013*map_31wks - 0.001*map_1 - 0.646*norm_wt - 0.829*over_wt - 1.127*obese - 0.059*matheight + 0.182*mat_over35 - 0.732*par2 - 0.783*par3 + 0.848*matsmok + 0.342*esshyp + 0.122*prev_gesthyp - 1.432*prev_gestdiab + 0.886*mat_nonwhite

34 weeks gestation: -1.64 + 0.020*map_34wks - 0.003*map_1 - 0.648*norm_wt - 0.861*over_wt - 1.203*obese - 0.060*matheight + 0.171*mat_over35 - 0.692*par2 - 0.745*par3 + 0.857*matsmok + 0.254*esshyp + 0.069*prev_gesthyp - 1.399*prev_gestdiab + 0.921*mat_nonwhite

36 weeks gestation: -1.67 + 0.011*map_36wks - 0.001*map_1 - 0.624*norm_wt - 0.819*over_wt - 1.158*obese - 0.059*matheight + 0.170*mat_over35 - 0.700*par2 - 0.754*par3 + 0.850*matsmok + 0.256*esshyp + 0.083*prev_gesthyp - 1.360*prev_gestdiab + 0.892*mat_nonwhite

Model 3

20 weeks gestation: -1.64 + 0.001*map_dev_20 + 0.003*map_1 - 0.625*norm_wt - 0.791*over_wt - 1.072*obese - 0.058*matheight + 0.182*mat_over35 - 0.756*par2 - 0.824*par3 + 0.850*matsmok + 0.445*esshyp + 0.207*prev_gesthyp - 1.445*prev_gestdiab + 0.857*mat_nonwhite

25 weeks gestation: -1.64 - 0.004*map_dev_25 + 0.004*map_1 - 0.626*norm_wt - 0.791*over_wt - 1.071*obese - 0.058*matheight + 0.186*mat_over35 - 0.758*par2 - 0.826*par3 + 0.849*matsmok + 0.462*esshyp + 0.218*prev_gesthyp - 1.438*prev_gestdiab + 0.853*mat_nonwhite

28 weeks gestation: -1.63 + 0.004*map_dev_28 + 0.003*map_1 - 0.630*norm_wt - 0.792*over_wt - 1.076*obese - 0.058*matheight + 0.183*mat_over35 - 0.756*par2 - 0.825*par3 + 0.850*matsmok + 0.427*esshyp + 0.194*prev_gesthyp - 1.411*prev_gestdiab + 0.877*mat_nonwhite

31 weeks gestation: -1.65 + 0.013*map_dev_31 + 0.003*map_1 - 0.626*norm_wt - 0.785*over_wt - 1.060*obese - 0.059*matheight + 0.182*mat_over35 - 0.747*par2 - 0.799*par3 + 0.841*matsmok + 0.341*esshyp + 0.122*prev_gesthyp - 1.432*prev_gestdiab + 0.887*mat_nonwhite

34 weeks gestation: -1.67 + 0.020*map_dev_34 + 0.003*map_1 - 0.625*norm_wt - 0.798*over_wt - 1.081*obese - 0.060*matheight + 0.172*mat_over35 - 0.720*par2 - 0.774*par3 + 0.844*matsmok + 0.254*esshyp + 0.069*prev_gesthyp - 1.400*prev_gestdiab + 0.921*mat_nonwhite

36 weeks gestation: -1.68 + 0.011*map_dev_36 + 0.002*map_1 - 0.613*norm_wt - 0.786*over_wt - 1.072*obese - 0.059*matheight + 0.171*mat_over35 - 0.719*par2 - 0.772*par3 + 0.842*matsmok + 0.256*esshyp + 0.082*prev_gesthyp - 1.361*prev_gestdiab 0.892*mat_nonwhite

Recalibration in SWS

Prediction models for preeclampsia at each gestational age were recalibrated for use in the SWS using the equation:

Logit(probability of preeclampsia in SWS) = $\alpha_{recal} + \beta_{recal}$ ($\boldsymbol{\beta X_{ALSPAC}}$)

where $\boldsymbol{\beta X_{ALSPAC}}$ are the parameters from the logistic regression in the ALSPAC cohort as detailed above and α_{recal} and β_{recal} represent the extent to which the intercept and the slope respectively of the regression model need to be altered for recalibration to the SWS cohort.

The values of α_{recal} and β_{recal} for each of the models at each gestational age are given in the table below.

Table: Recalibration parameters for each of the prediction models for preeclampsia

**Gestational age**	**Model**	α_{recal}	β_{recal}
**Initial model**	1	-1.55 (-2.29, -0.82)	0.73 (0.51, 0.95)
**20 weeks**	2	-1.38 (-2.08, -0.68)	0.75 (0.54, 0.95)
	3	-1.38 (-2.08, -0.68)	0.74 (0.54, 0.95)
**25 weeks**	2	-1.38 (-2.04, -0.72)	0.73 (0.54, 0.93)
	3	-1.39 (-2.05, -0.73)	0.73 (0.53, 0.92)
**28 weeks**	2	-1.03 (-1.78, -0.28)	0.87 (0.66, 1.09)
	3	-1.03 (-1.79, -0.27)	0.87 (0.65, 1.09)
**31 weeks**	2	-0.85 (-1.54, -0.16)	0.93 (0.72, 1.14)
	3	-0.85 (-1.54, -0.16)	0.93 (0.72, 1.14)
**34 weeks**	2	-0.61 (-1.20, -0.01)	1.00 (0.80, 1.19)
	3	-0.61 (-1.21, -0.01)	0.99 (0.79, 1.19)
**36 weeks**	2	-0.53 (-1.29, -0.23)	0.98 (0.77, 1.18)
	3	-0.54 (-1.28, 0.19)	0.97 (0.77, 1.17)

References

(1) Harrell FE, Jr. *Regression modeling strategies: With applications to linear models, logistic regression and survival analysis.* New York: Springer; 2001.

(2) Tilling K, Sterne JAC, Wolfe CDA. Multilevel growth curve models with covariate effects: Application to recovery after stroke. *Stat Med*. 2001;20:685-704.

(3) Pan HQ, Goldstein H. Multi-level models for longitudinal growth norms. *Stat Med*. 1997;16:2665-2678
