# Supplementary material for: Antenatal blood pressure for prediction of pre-eclampsia, preterm birth, and small for gestational age babies: development and validation in two general population cohorts
Source: BMJ. 2015 Nov 17;351:h5948. doi: 10.1136/bmj.h5948 (PMC4647185; doi:10.1136/bmj.h5948)
Supplement: Supplementary file 3 [file macc026935.ww3_default.pdf]

Appendix 3: Supplementary figures A and B [posted as supplied by author]

Figure A ROC curves at 28 weeks gestation for prediction of preeclampsia in ALSPAC and SWS*

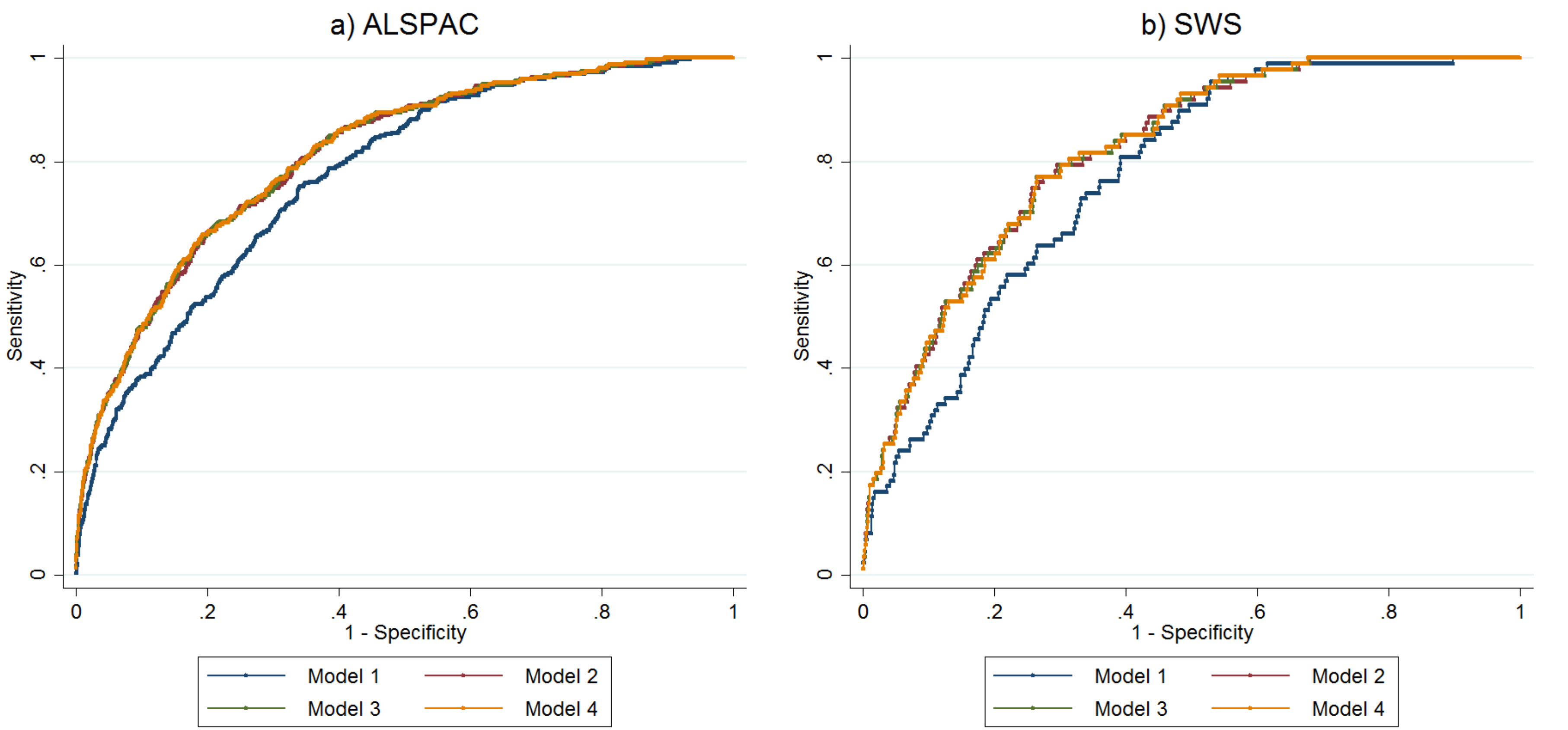

* Model 1 includes maternal covariates (pre-pregnancy BMI, height, age over 35, parity, smoking, essential hypertension, previous gestational hypertension, diabetes, previous gestational diabetes, non-white ethnicity) and MAP at the first visit; Model 2 is as Model 1 plus the observed value of MAP at 28 weeks; Model 3 is as Model 1 plus the deviation of the observed value of MAP from the normogram prediction at 28 weeks; Model 4 is as Model 1 plus both the observed value of MAP at 28 weeks and the deviation of the observed value of MAP from the normogram prediction at 28 weeks

Figure B Expected numbers of women allocated to the low and high risk groups for preeclampsia from a cohort of 1000 women based on testing sequentially using Model 1 at booking followed by Model 2 at subsequent visits with a threshold of 0.013 at each gestational age up to 31 weeks*

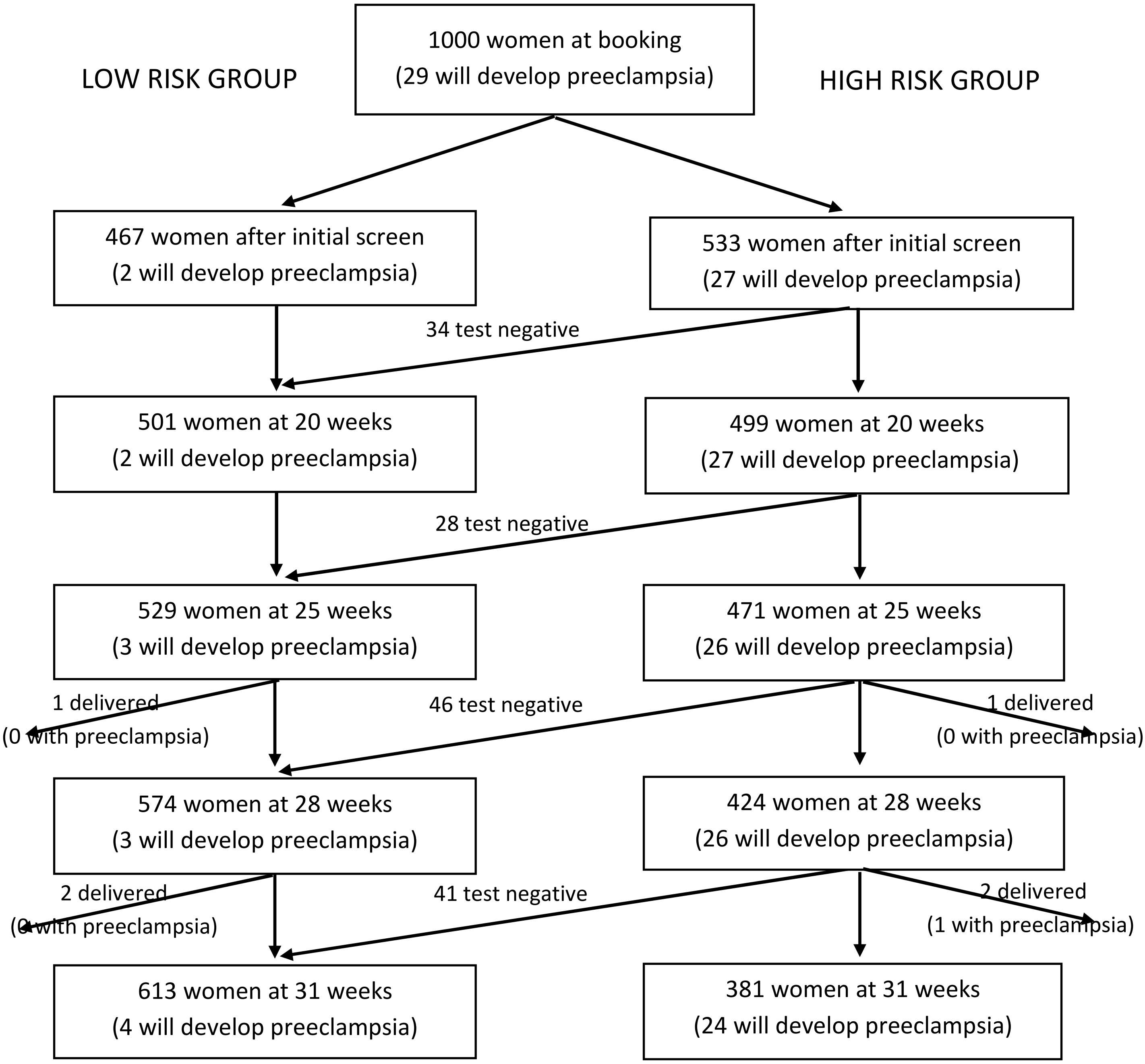

* After the initial screen, only the high risk group are included in subsequent screening tests. To obtain these numbers we determined the average threshold giving a 95% sensitivity at each stage in the Avon Longitudinal Study of Parents and Children (including the high risk group only after the initial screen), and applied this threshold in the Southampton Women's Survey.
Model 1 includes maternal covariates and MAP at the first visit; Model 2 additionally includes MAP at the gestational age of testing.
